# Genetic diversity and antagonistic properties of *Trichoderma* strains from the crop rhizospheres in southern Rajasthan, India

**DOI:** 10.1038/s41598-024-58302-5

**Published:** 2024-04-14

**Authors:** Prashant P. Jambhulkar, Bhumica Singh, M. Raja, Adnan Ismaiel, Dilip K. Lakshman, Maharishi Tomar, Pratibha Sharma

**Affiliations:** 1 Department of Plant Pathology, College of Agriculture, Rani Lakshmi Bai Central Agricultural University (RLBCAU), Jhansi, Uttar Pradesh 284003 India; 2 Agricultural Research Station, Banswara, Rajasthan 327001 India; 3 Department of Plant Pathology, Sri Karan Narendra Agriculture University, Jobner-Jaipur, Rajasthan 303328 India; 4 Sustainable Agricultural Systems Laboratory, USDA-ARS, Beltsville, MD 20705 USA; 5 ICAR-Indian Grassland and Fodder Research Institute, Jhansi, 284003 India

**Keywords:** Microbiology, Molecular biology, Plant sciences

## Abstract

There are fewer studies on *Trichoderma* diversity in agricultural fields. The rhizosphere of 16 crops was analyzed for *Trichoderma* species in 7 districts of Rajasthan state of India. Based on DNA sequence of translation elongation factor 1α *(tef-1α*), and morphological characteristics, 60 isolates were identified as 11 species: *Trichoderma brevicompactum*, species in *Harzianum clade* identified as *T. afroharzianum*, *T. inhamatum*, *T. lentiforme*, *T. camerunense*, *T. asperellum*, *T. asperelloides*, *T. erinaceum*, *T. atroviride*, *T. ghanense*, and *T. longibrachiatum. T. brevicompactum* is the most commonly occurring strain followed by *T. afroharzianum*. No new species were described in this study. *T. lentiforme*, showed its first occurrence outside the South American continent. The morphological and cultural characteristics of the major species were observed, described, and illustrated in detail. The isolates were tested for their antagonistic effect against three soilborne plant pathogens fungi: *Sclerotium rolfsii, Rhizoctonia solani,* and *Fusarium verticillioides* in plate culture assays*.* One of the most potent strains was *T. afroharzianum* BThr29 having a maximum in vitro inhibition of *S. rolfsii* (76.6%), *R. solani* (84.8%), and *F. verticillioides* (85.7%). The potential strain *T. afroharzianum* BThr29 was also found to be efficient antagonists against soil borne pathogens in in vivo experiment. Such information on crop selectivity, antagonistic properties, and geographic distribution of *Trichoderma* species will be beneficial for developing efficient *Trichoderma*-based biocontrol agents.

## Introduction

Species in the fungal genus *Trichoderma* (Hypocreales, Ascomycota) are known for rapid growth and high-stress tolerance. Many of the *Trichoderma* species have a cosmopolitan distribution and inhabit a wide range of ecological niches such as soil, decayed wood, endophytes of plants, mushroom compost, and marine habitats^1,2^. Currently, there are about 500 species based on legitimate names in the Mycobank ((https://www.mycobank.org.) the majority of them have been described during the past two decades based on multi-locus phylogeny analyses of DNA sequences^3–7^. Native *Trichoderma* strains from crop rhizosphere are likely to be more effective biocontrol agents as they are in close contact with plant roots and have higher chances to establish endophytic relationships and exert the benefits associated with the relationship such as induced resistance to pathogens and enhance plant growth^8^. The occurrence and prevalence of *Trichoderma* in different ecological niches have contributed to the evolution of the species and genetic and metabolic diversities. This immense ecological importance of *Trichoderma* spp. and plant association makes it pertinent to understand their biodiversity in the crop rhizosphere.

Taxonomic classification of *Trichoderma* species remained obscure until 1969, about 200 years after the genus name *Trichoderma* was introduced. Rifai^9^ used morphological characters plus characters from the teleomorphs and distinguished nine aggregate species and emphasized that each aggregate species could harbour more species. Bissett^10–13^ revised Rifai’s aggregate species by looking at them as sections and recognized four sections and 27 biological species. Bissett rarely used characters from teleomorphs. In the early 1990s phylogenies based on DNA sequencing data started and revealed some important achievements in *Trichoderma* taxonomy. Phylogeny based on sequences of the ribosomal large subunit revealed that *Gliocladium* comprised three paraphyletic groups distinct in morphology and that *G. virens* is *Trichoderma virens*. Rehner and Samuels^14^ also revealed that phylogenetically *Trichoderma* and *Hypocrea* are indistinguishable, and they both fit into identical clades. Later, inaccuracies in *Trichoderma* taxonomy based on morphological characters alone were shown in the study of Kullnig et al.^15^, which confirmed that the phylogenies based on sequencing data are indispensable. This led to an explosion in the number of *Trichoderma* species. Currently, the DNA sequences of ribosomal internal transcribed spacer (ITS), translation elongation factor *1α* (*tef-1α*), and RNA polymerase subunit2 (*rpb2*) are used for the identification of new species when used in multi-locus phylogeny^16^.

Previously, researchers explored crop rhizospheres in different parts of India. Kumar et al.^17^ characterized the isolates of *Trichoderma* spp. from south Andaman for their cultural, morphological, and antagonistic activity against soilborne and foliar pathogens such as *Sclerotium rolfsii*, *Colletotrichum gloeosporioides,* and *C. capsici*. Kamala et al.^18^ isolated 193 *Trichoderma* strains from cultivated fields of the Indian region of the Indo-Burma Biodiversity hotspot. Meena and Meena^19^ did morphological and physiological characterization of eight *Trichoderma* spp isolates collected from cluster bean fields in western Rajasthan. They studied their antagonism against *Macrophomina phaseolina, Fusarium oxysporum,* and *Sclerotinia sclerotiorum*. Rai et al.^20^ collected *Trichoderma* spp. from tomato rhizosphere and characterized it phenotypically, biochemically, and genetically to screen the most efficient *Trichoderma* spp. against pathogens causing disease in tomato crops. Overlapping morphological characters of *T. pseudokoningii* and *T. longibrachiatum* were characterized by Prameeladevi et al.^21^ using ITS and *tef-1α* genes and confirmed that isolates identified as *T. pseudokoningii* in India are genuinely *T. longibrachiatum*. The value of *Trichoderma* spp. depends on their applicability in different crops growing in varied ecozones. Therefore, collecting *Trichoderma* isolates from different crop rhizosphere is essential, which will help select the most efficient antagonistic strains of *Trichoderma* spp. Moreover, the southern part of Rajasthan was untouched to explore genetic diversity and utilize the potential of its *Trichoderma* spp. and strains; thus, it was necessary first to isolate, identify and characterize the isolates of *Trichoderma*. Growth media markedly affects the colony formed^22^. Various studies were conducted to evaluate different culture media for the growth of *T. harzianum*^23,24^, Several selective media for *Trichoderma* spp. were previously described^25,26^. In the present study, we emphasized on selection of the appropriate growth media to facilitate efficient isolation of potential *Trichoderma* spp. from the soil.

**Figure 1:**
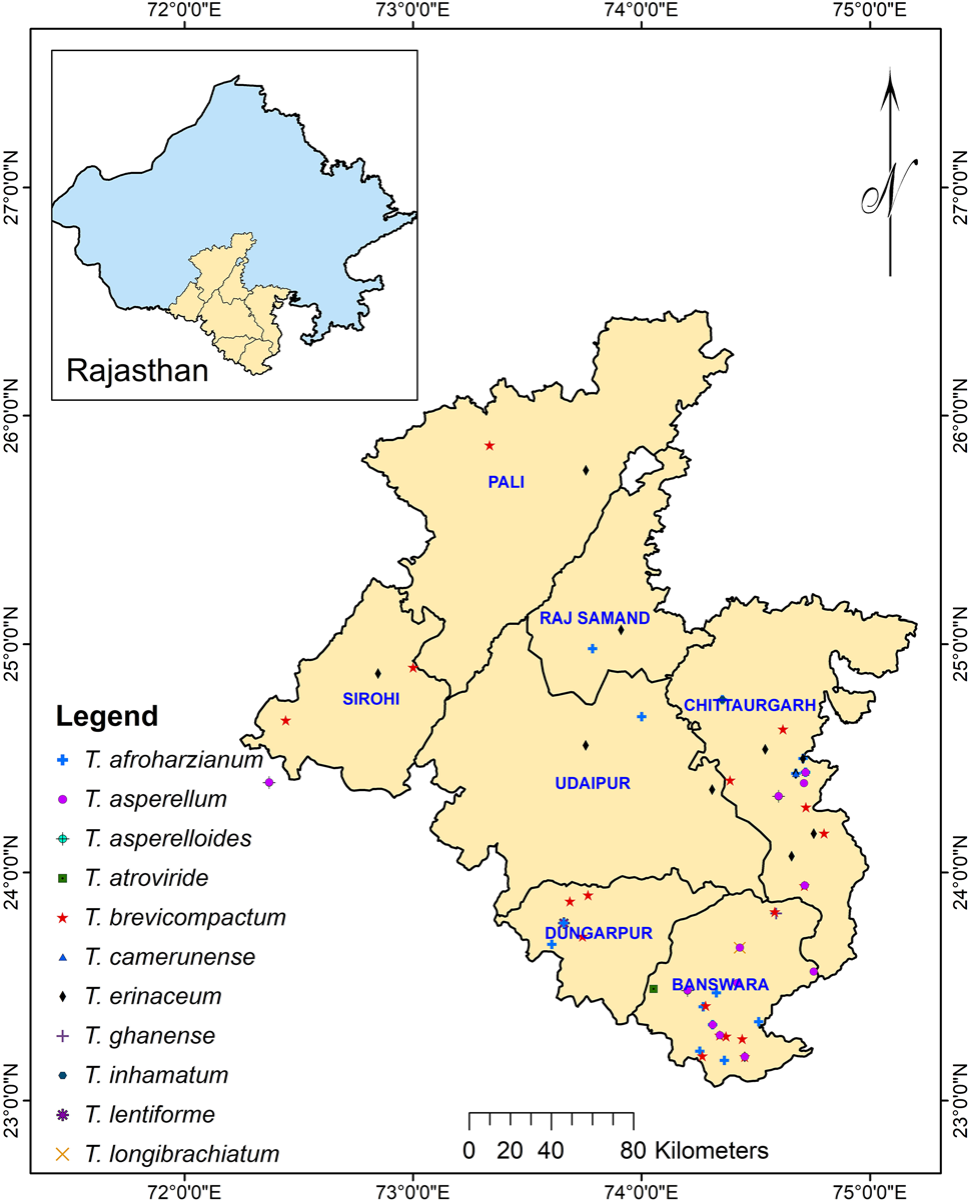
Map of southern Rajasthan showing different locations of *Trichoderma* isolate collection. Arc GIS 10.1 platform (ESRI Inc., United States) was used for preparing the map for the study area and sample location sites.

To utilize the full potential of the *Trichoderma* species in specific applications, precise identification and characterization of this fungus are vital^27^. The present study was carried out to characterize and identify *Trichoderma* species isolated from 16 different crop rhizosphere soils in southern Rajasthan by using morphological characterization and sequence analysis of translation elongation factor (*tef-1α*) section that encompasses few introns (Fig. 1). Potentiality of the *Trichoderma* strains was also evaluated in in vitro and in vivo assays.

## Result

**Figure 2:**
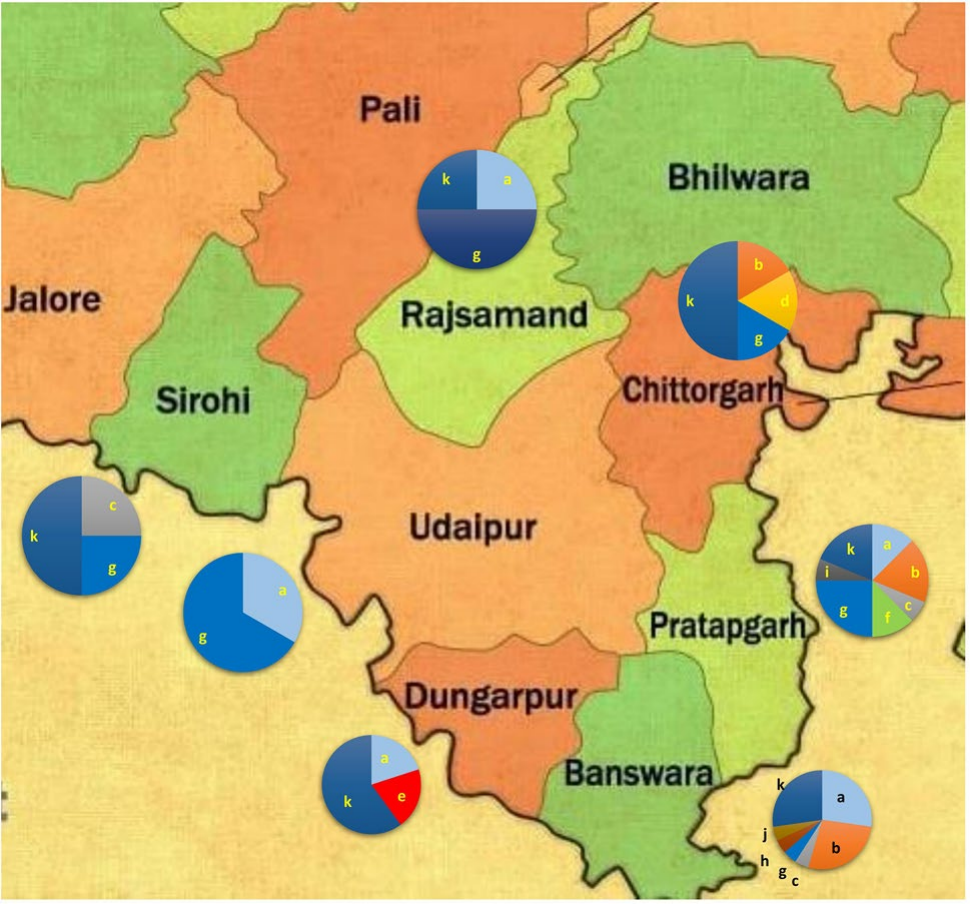
Distribution of *Trichoderma* species in the different districts of southern Rajasthan, where, (**a**) *T. afroharzianum*; (**b**) *T. asperellum*; (**c**) *T. asperelloides*; (**d**) *T. inhamatum*; (**e**) *T. lentiforme*; (**f**) *T. camerunense*; (**g**) *T. erinaceum*; (**h**) *T. atroviride*; (**i**) *T. ghanense*; (**j**) *T. longibracheatum*; (**k**) *T. brevicompactum.*

The isolation rate of *Trichoderma* isolates from 273 soil samples was 22%. Details of the strains isolated from two agroclimatic zones namely, Zone IVA-Sub-humid southern plains and IVB-Humid southern plains of southern Rajasthan in India, are given in Supplementary table 1. The isolates were identified using morphological characters and the DNA sequences of *tef-1α* section that encompass a few introns. The total 60 isolates were classified into 11 species: *Trichoderma afroharzianum* (11), *T. asperellum* (10), *T. asperelloides* (3), *T. inhamatum* (1), *T. camerunense* (2), *T. erinaceum* (11), *T. lentiforme* (1), *T. atroviride* (1), *T. ghanense* (1), *T. longibrachiatum* (1), *T. brevicompactum* (18). These eleven species are prevalent in the crop rhizosphere of southern Rajasthan (Fig. 2).

### Phylogeny

**Figure 3:**
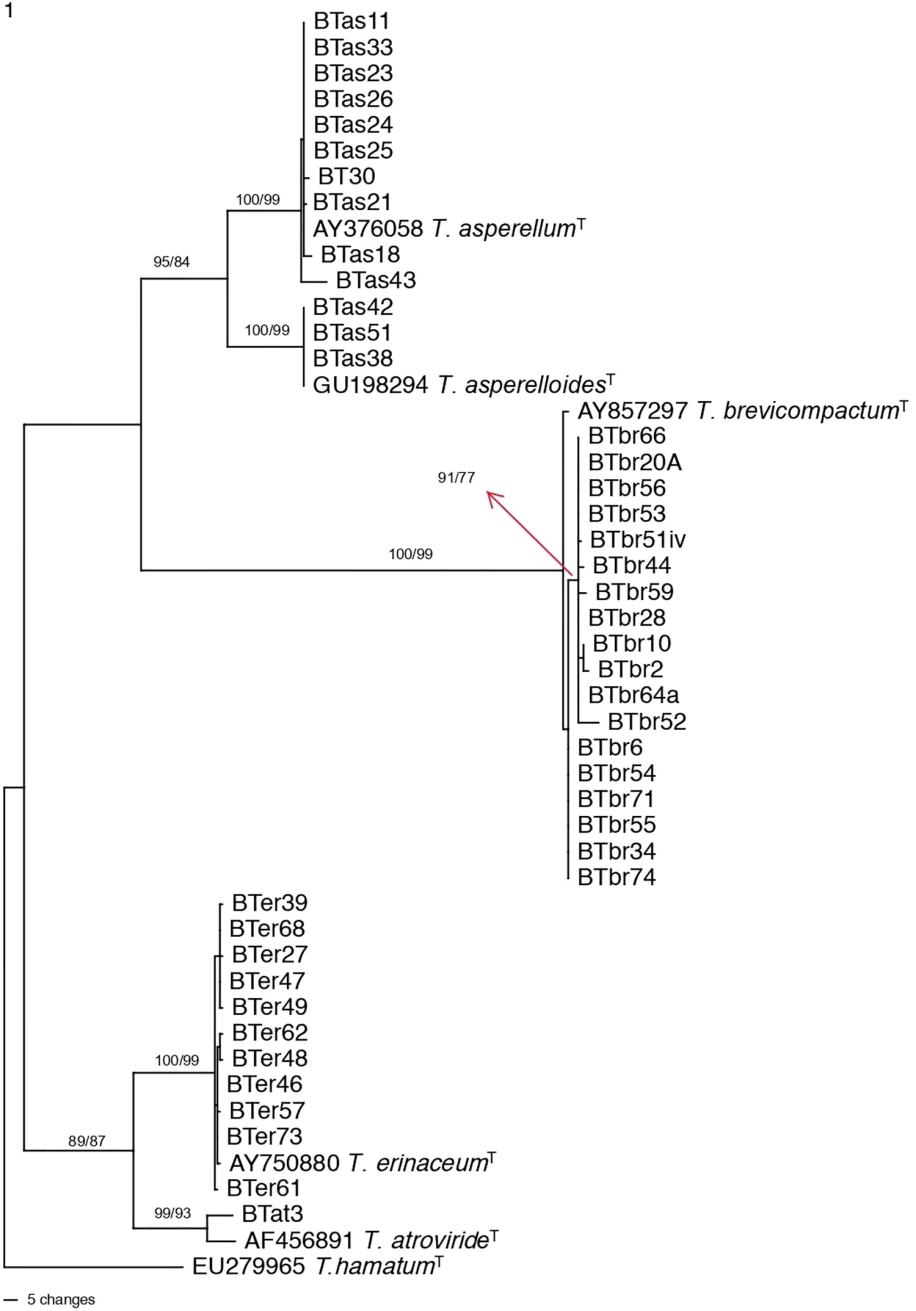
Phylogenetic relationships of *Trichoderma* section Trichoderma isolates from India. One of most parsimonious trees generated from *tef-1α* sequence data. Bootstrap values are given (Parsimony/Maximum Likelihood) on the branches. ^T^ at the end of leaf label refers to type species sequence. ^B^ at the end of leaf label refers to culture used in biocontrol. Tree was rooted to *T. hamatum*.

Due to high variability of *tef-1α* section and the difficulty of aligning all the species of the *Trichoderma* genus together, we divided the alignment file into two files, one representing species within *Trichoderma* section and *T. brevicompactum*. The other comprising species in the *T. harzianum* complex clade and *T. longibrachiatum* section. The first file contained 54 taxa, including five reference sequences for five specific type species and one outgroup taxa. The tree in Fig. 3 shows that there were 18 isolates (BTbr66, BTbr20A, BTbr56, BTbr53, BTbr51iv, BTbr44, BTbr59, BTbr28, BTbr10, BTbr2, BTbr64a, BTbr52, BTbr6, BTbr54, BTbr71, BTbr55, BTbr34, and BTbr74) clustered with the type species of *T. brevicompactum* (AY857297) with high Bootstrap values, clearly indicating that the 18 isolates are *T. brevicompactum*.

Three taxa (BTas41, BTas51, and BTas38) formed a highly supported clade with type species of *T. asperelloides* (GU198294). Another 10 strains (BTas1, BTas33, BTas23, BTas26, BTas24, BTas25, BT30, BTas21, BTas18, and BTas43) clustered with the type species of *T. asperellum* with high bootstrap value. The *T. asperellum* and *T. asprelloides* clades have sister-relationship with a high bootstrap value of 84. One isolate (BTat3) formed a clade with the type species sequence of *T. atroviride* which confirms the identity of the isolate as *T. atroviride*. *T. atroviride is* known to be dominant in North America and Europe but rare in tropical regions and well known for biocontrol applications. Finally, 11 strains (BTer39, BTer68, BTer27, BTer47, BTer49, BTer62, BTer48, BTer46, BTer57, BTer73, and BTer61) clustered with the type species of *T. erinaceum* (AY750880), clearly indicating the identity of the 11 strains as *T. erinaceum. T. erinaceum* has sister relationship *with T. atroviride* with high bootstrap values (89/87).

**Figure 4:**
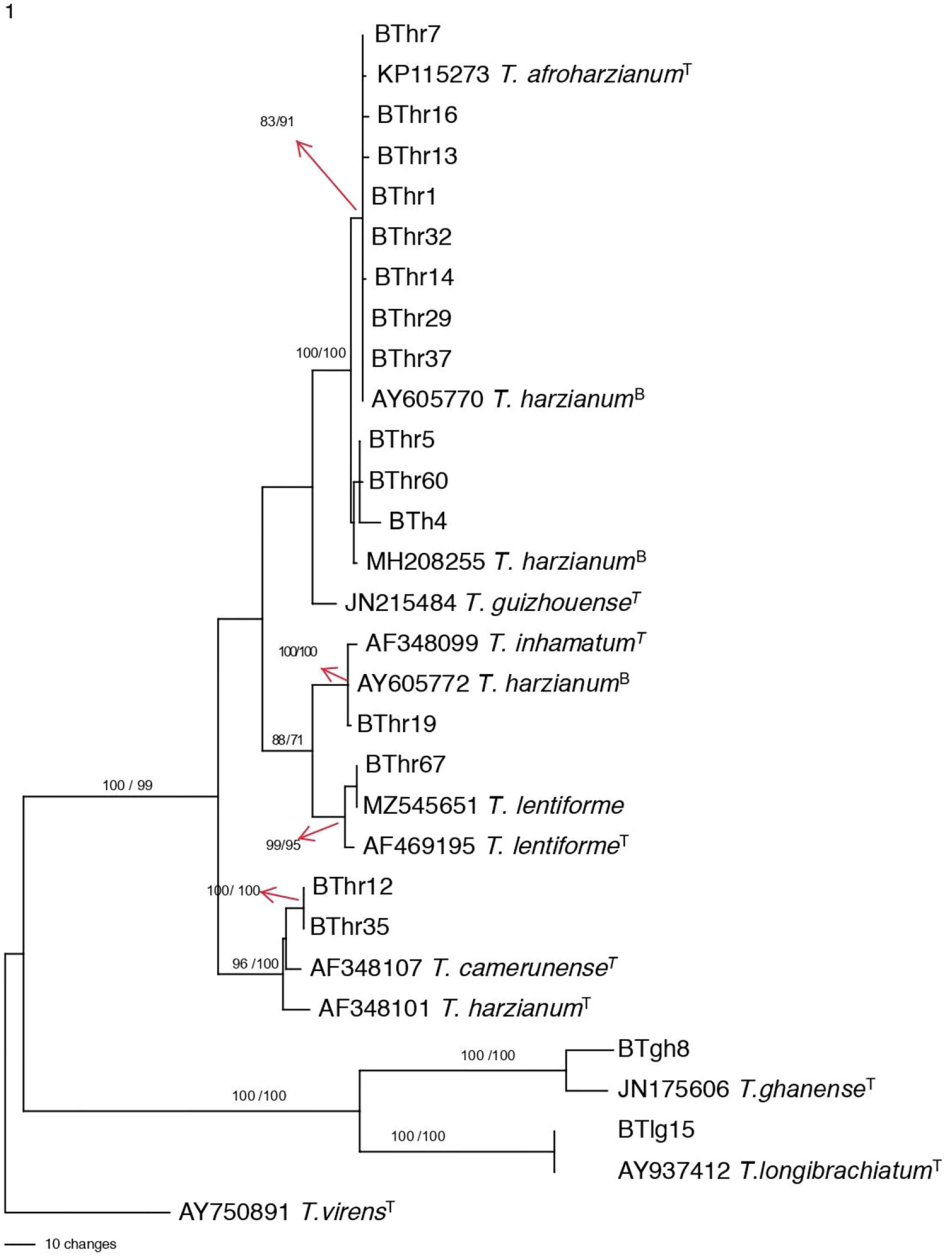
Phylogenetic relationships of Trichoderma harzianum complex species and section Longibrachiatum isolates from India. One of most parsimonious trees generated from tef-1α sequence data. Bootstrap values are given (Parsimony/Maximum Likelihood) on the branches. ^T^ at the end of leaf label refers to type species sequence. ^B^ at the end of leaf label refers to culture used in biocontrol. Tree was rooted to *T. virens.*

In the second phylogenetic tree Fig. 4 11 strains (BThr7, BThr16, BThr13, BThr1, BThr32, BThr14, BThr29, BThr37, BThr5, BThr16, and BTh4) formed a clade with type strain for *T. afroharzianum* (KP115273) and another reference sequence deposited in GenBank as *T. harzianum* (AY605770)*.* The clade of 12 isolates had high bootstrap values of 83 and 91 for parsimony and maximum likelihood analyses, respectively. *T. afroharzianum* sequence was identical to sequences of five of the isolates under the study and differed from the other five isolates by one base, clearly indicating that the clade of 12 isolates represents an all *T. afroharzianum* clade*.* Another isolate BThr19 formed a highly supported clade with the type strain species of *T. inhamatum* (AF348099), which was obtained from Thailand. One isolate BThr67 clustered with the type species sequence for *T. lentiforme.* The last two strains of the *T. harzianum* clade (BThr12 and BThr35) formed a clade with high bootstrap value 96 /100 for parsimony and maximum likelihood analyses, respectively with type species of *T. camerunense* which is a dominant species of Harzianum clade species in Africa. Of the two strains from the Longibrachiatum section one (BTlg15) formed a clade with type species *of T. longibrachiatum* with bootstrap support of 100, clearly indicating that BTlg15 is *T. longibrachiatum.* The other strain BTgh8 formed a clade with the type strain of *T. ghanense* with high support. This indicates that BTgh8 is *T. ghanense*.

### Relative abundance of different *Trichoderma* species in different crops

**Figure 5:**
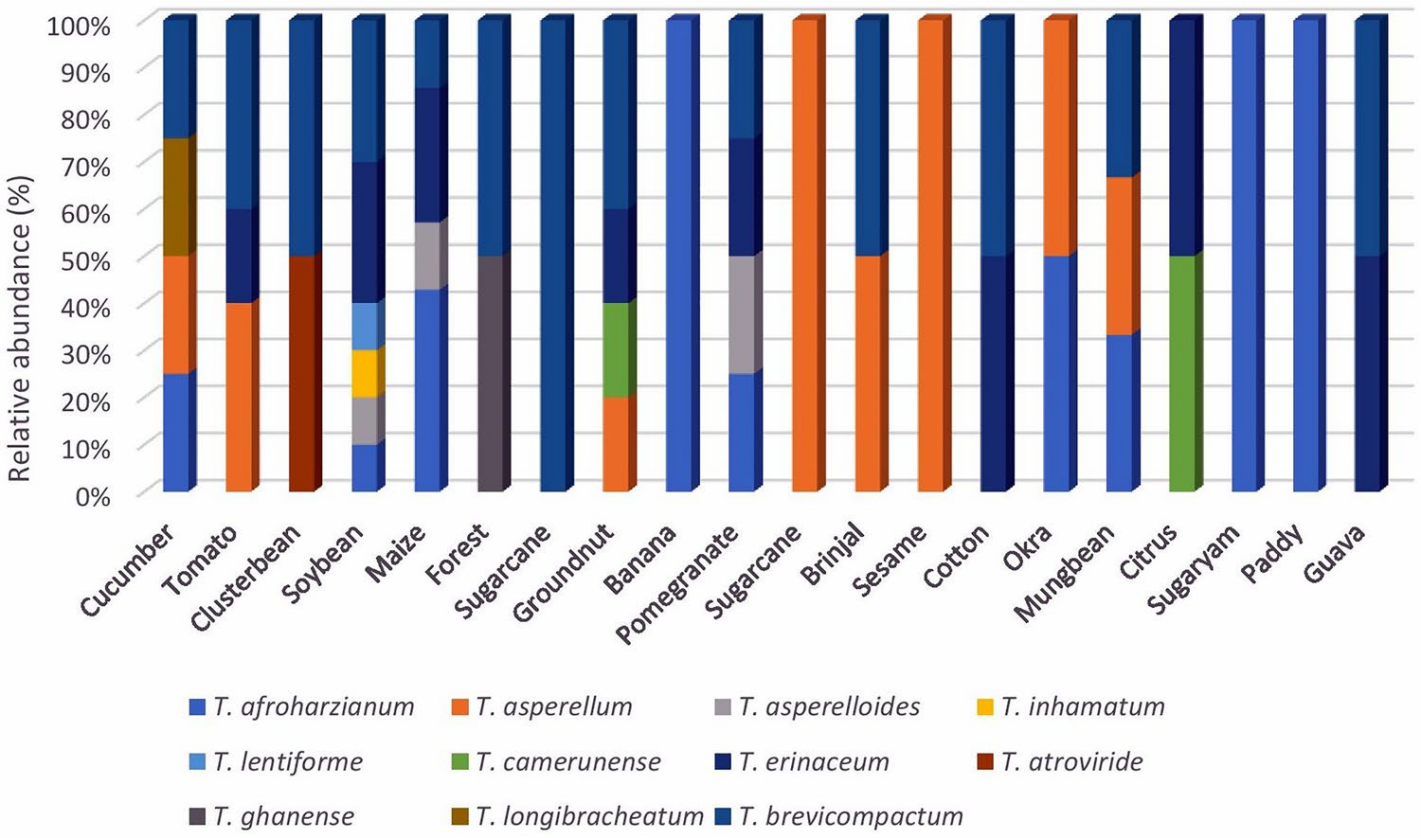
Relative abundance of *Trichoderma* species in crop rhizosphere. Stacked bars represent the number of individual strains of *Trichoderma* in different crop rhizosphere.

The *Trichoderma* strains were isolated from the rhizosphere of 16 crops cultivated in fields of southern Rajasthan. The 60 isolates are classified into eleven species that have varied abundance in different crop rhizosphere. It was observed that a maximum of 10 *Trichoderma* strains, three *T. erinaceum*, three *T. brevicompactum,* one each species of *T. afroharzianum, T. asperelloides, T. inhamatum*, and *T. lentiforme* were isolated from the soybean crop rhizosphere. This was followed by maize rhizosphere, from which seven strains of *Trichoderma* were isolated, including three strains of *T. afroharzianum*, two strains of *T. erinaceum,* and one each strain of *T. asperelloides*, and *T. brevicompactum* (Fig. 5). Eighteen strains of *T. brevicompactum* occurred in 12 crop rhizospheres, while eleven strains each of *T. afroharzianum* and *T. erinaceum* were isolated from crop rhizosphere of nine and eight crops, respectively. *T. inhamatum*, *T. ghanense, T. longibrachiatum,* and *T. atroviride* were found in individual crop rhizospheres. The relative dominance values calculated showed that the genus *Trichoderma* is not dominant in the soil samples. The dominance value of *T. brevicompactum, T. erinaceum, T. afroharzianum, T. asperellum* was 0.016, 0.012, 0.01, and 0.008, respectively, which was very low as compared to the standard dominance value (> 0.02).

### Effect of different media on growth and sporulation of different *Trichoderma* spp.

**Figure 6:**
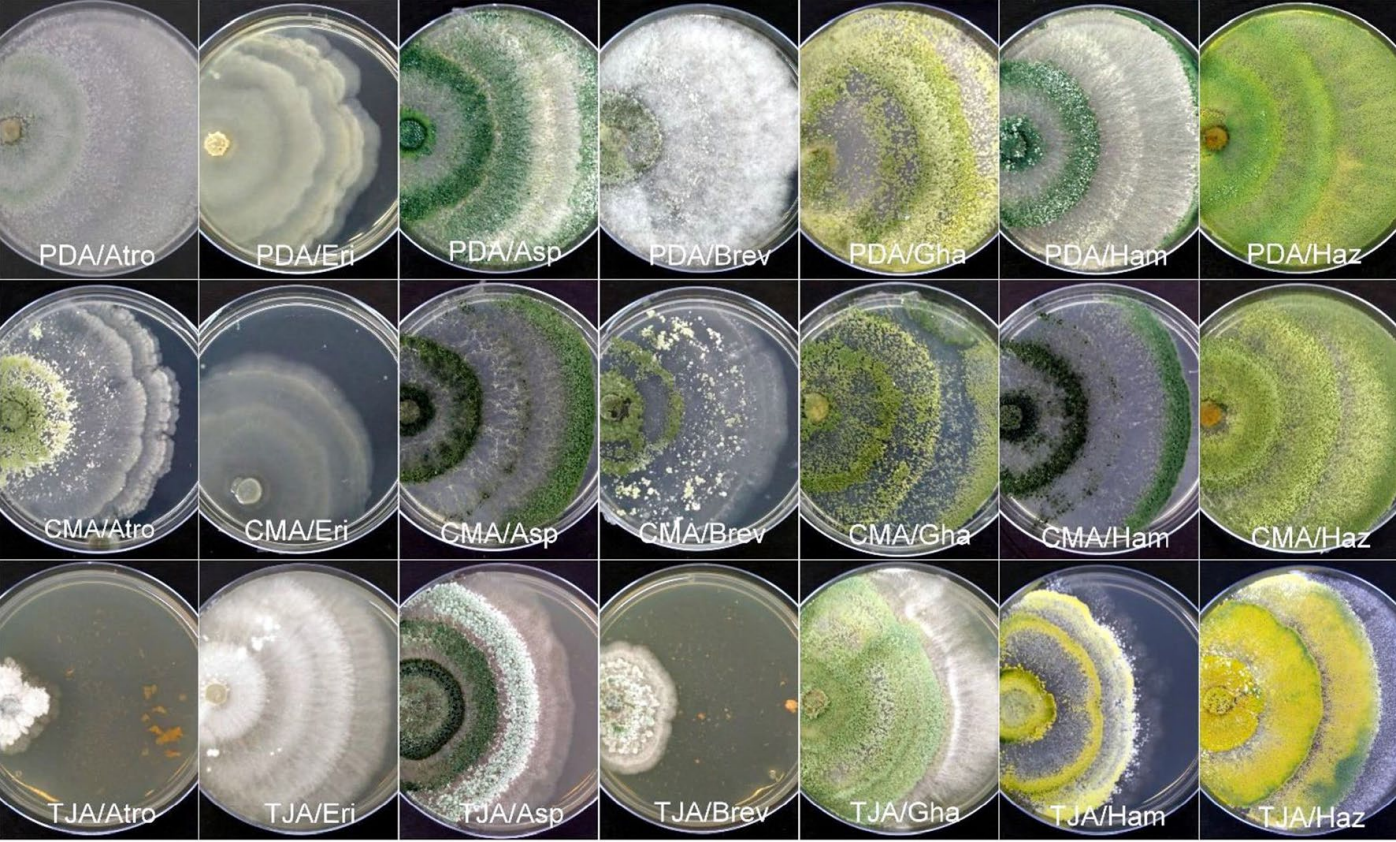
Plates showing growth of 7 major species of *Trichoderma* on 3 different growth media (PDA, CMA, and TJA).

**Figure 7:**
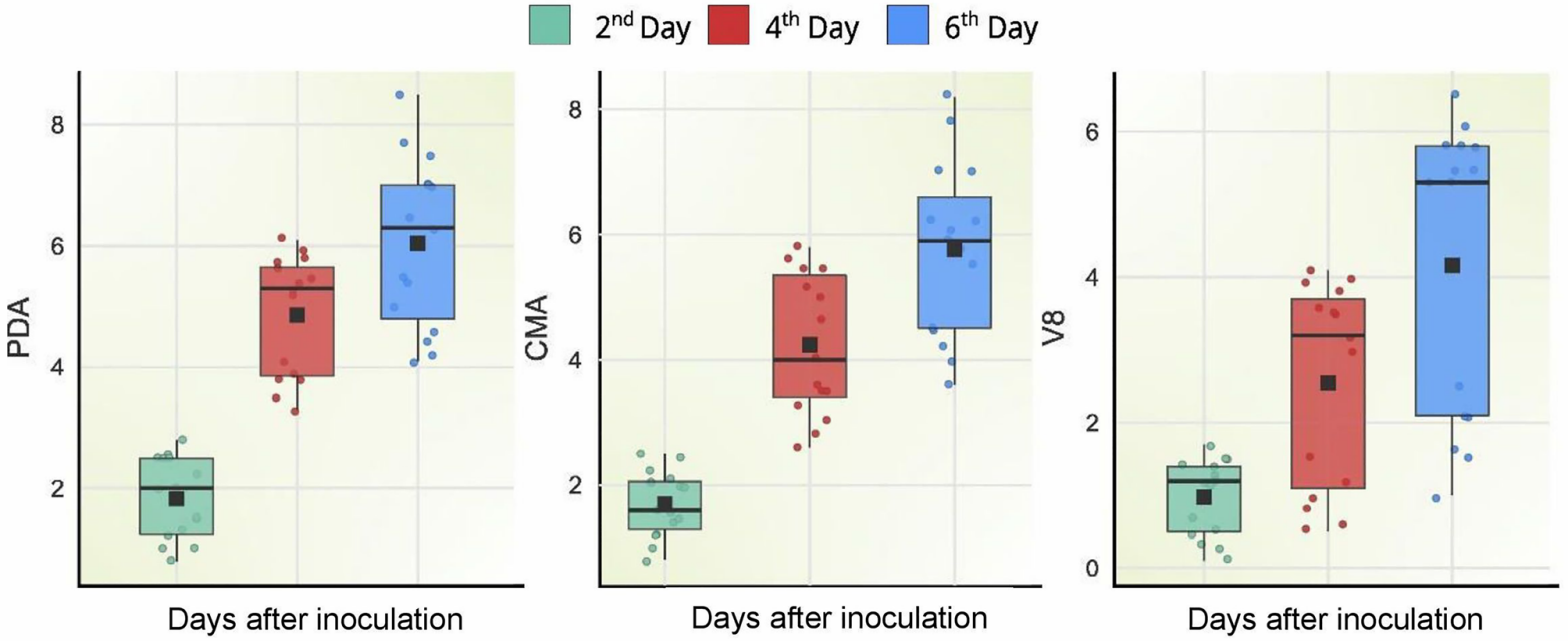
Box plot representing growth of *Trichoderma* spp. in growth media (PDA, CMA, and TJA) at 2, 4, and 6 days after inoculation.

Representative isolates of different *Trichoderma* species showed different growth patterns in different media. Among all the cultured species, *T. harzianum* (= *T. afroharzianum*)*, T. asperellum,* and *T. erinaceum* had the fastest growth in PDA medium (Fig. 6). Box plot analysis (Fig. 7) of three media that actively support the growth of *Trichoderma* strains revealed that at 2 days of incubation *T. asperellum, T. erinaceum,* and *T. afroharzianum* cover 2.2, 2.6, and 2 cm on PDA media which grows to 5.7, 5.3, and 5.8 cm after 4 days of incubation (Supplementary table 2). At 6 days of incubation, all three species of *Trichoderma* cover 7 cm growth on PDA media. The CMA medium is the next best medium for the growth of *Trichoderma* spp. The *T. asperellum, T. erinaceum*, and *T. afroharzianum* grow to 2.1, 2, and 2 cm on CMA media after 2 days of growth which rose to 4, 5.23, and 5.63 cm at 4 days of incubation (Supplementary table 2). At 6 days of incubation, *T. asperellum* showed a growth of 7 cm, and *T. erinaceum* 6.1 cm. *T.afroharzianum* attain a maximum growth of 7 cm. TJA media poorly support the growth of *Trichoderma* spp.

### Morphological characterization

In this study, we have reported seven species of *Trichoderma* prevalent in the rhizosphere of cultivated fields of southern Rajasthan. The strains were confirmed with the general morphological characters as described below.

### *T. ghanense*

**Figure 8:**
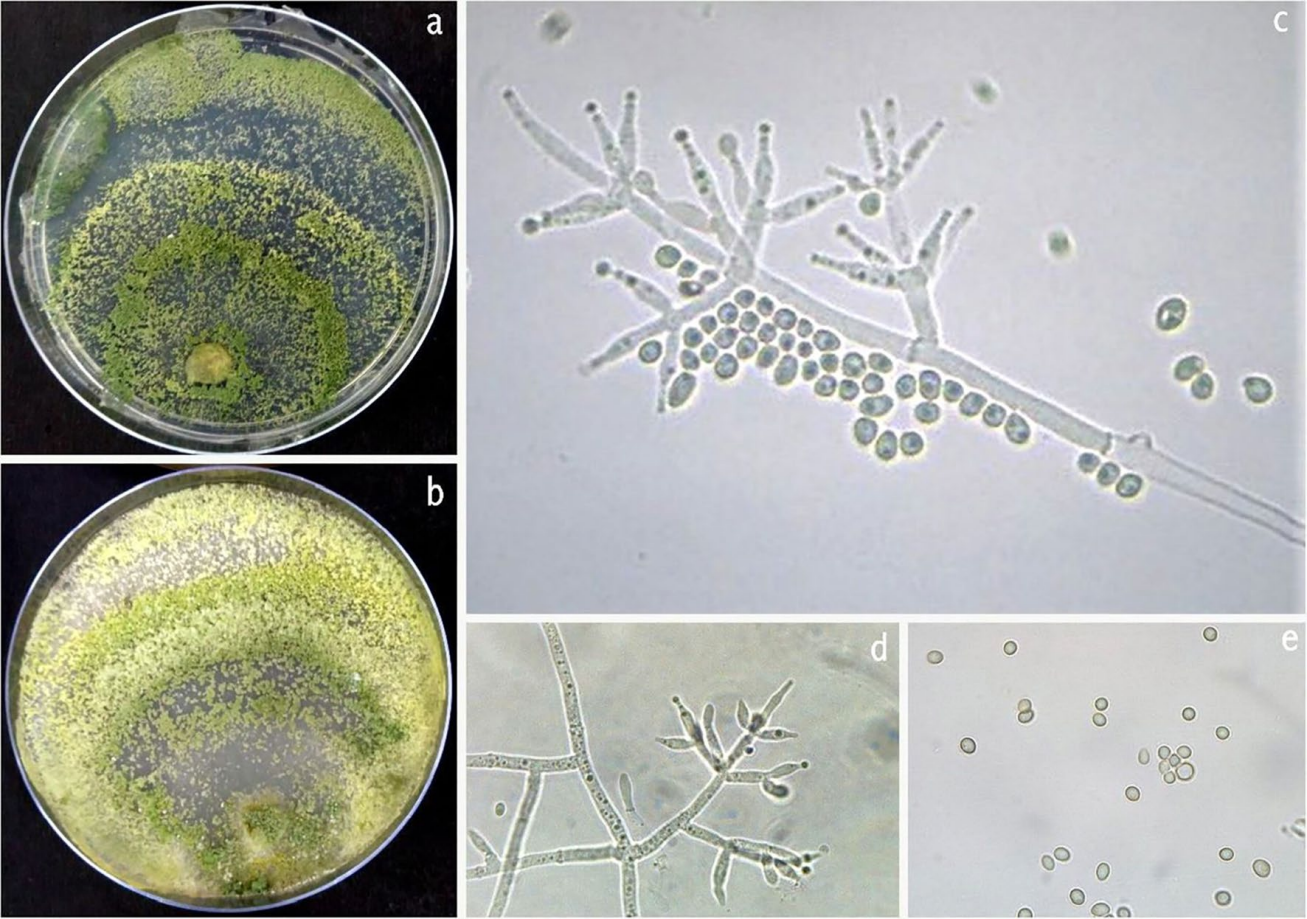
*Trichoderma ghanense*. (**a**, **b**) Cultures at 27 °C, 7d (a. on PDA; b. on CMA); (**c**, **d**) Conidiophores and phialides; **(e)** Conidia (PDA, 2d).

On the PDA medium after 72 h, the colony radius was 38–41 mm, mycelium covering the plate after 5 d at 27 °C. Colony dense, circular, not finely zonate with aerial hyphae more abundant at the colony center, growing fertile. Conidiation starts after two days of incubation, effuse in aerial hyphae or small granules spread in the entire plate but absent from the colony center, and turn green (Fig. 8). On the back side of the plate appears dark brown pigmentation. Numerous conidiophores, *Trichoderma*-like, branches paired or unpaired, at right or acute angles with the main axis, not or re-branched once. Paired phialides, lageniform, and sometimes ampulliform, are reported to have tuberculate conidia, but in microscope preparations, many or most conidia do not have visible tubercles, and typically only one or a few tubercles are seen on individual conidia. Conidia of Banswara strain (BTgh08) were considerably smaller (1.74 ± 0.15 × 1.45 ± 0.10 µm). *T. ghanense* is typically a soil species and has not been linked to a teleomorph. This strain was isolated from Peepalkhunth forest soil N 23° 49′ 9.9″ E 74° 35′ 21.8″.

### *T. afroharzianum*^9^

**Figure 9:**
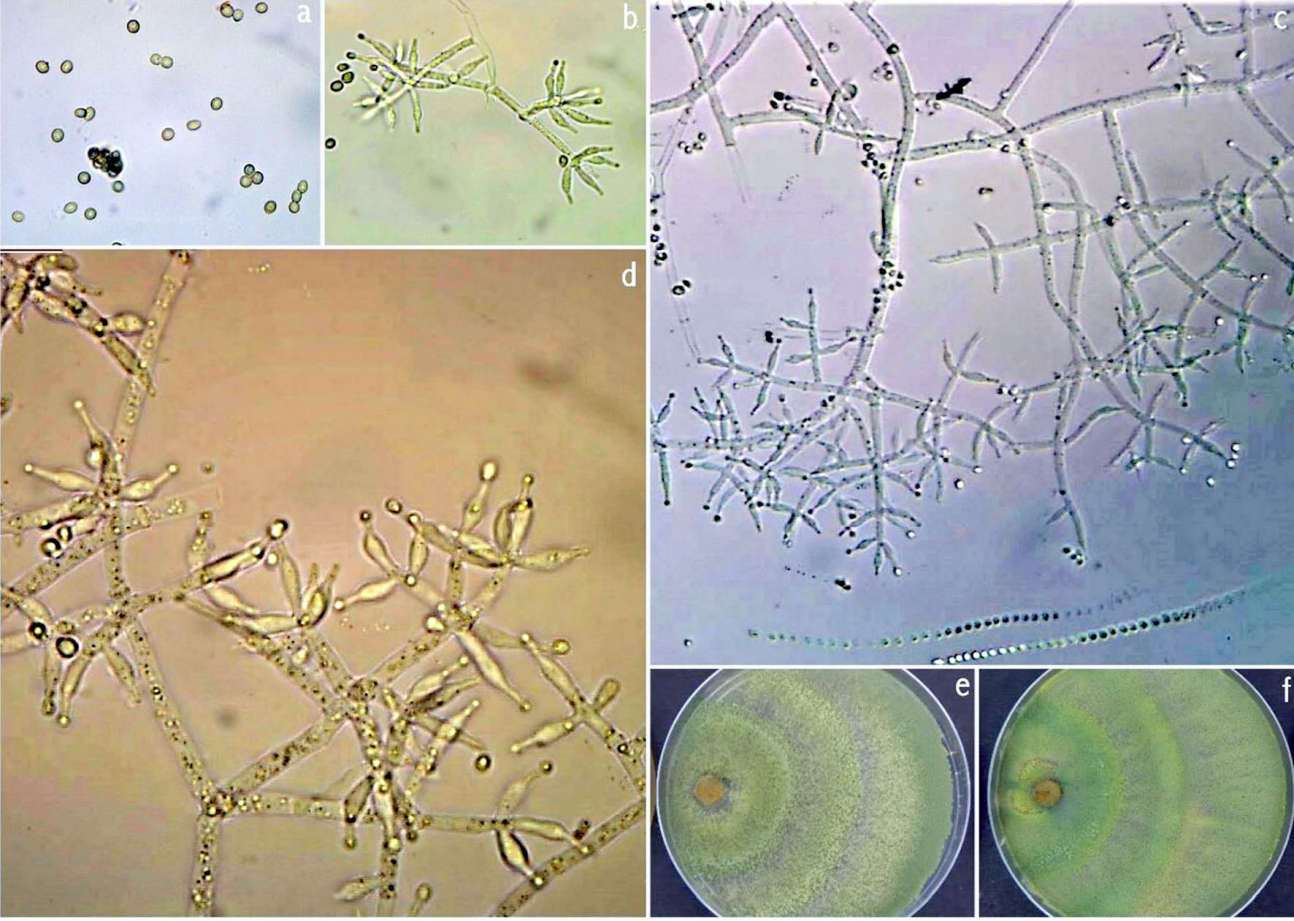
*Trichoderma afroharzianum*. **(a)** Conidia (PDA, 2d), (**b**–**d**) Conidiophores and phialides, (**e**–**f**) Cultures at 27 °C, 7d (e. on CMA; f. on PDA).

Colony on PDA medium in 72 h covers 45–50 mm, mycelium covers plate at 6th day after inoculation at 27 °C. The conidia appear green from the 2nd day onwards. The conspicuous concentric rings appear on the scale. The colony on PDA appears powdery, light pale green, and later gray-green. Light to dark brown pigmentation appeared at the bottom of the plate. A typical green mat of conidia with concentric rings also appeared on CMA. The different isolates had two types, 1. Brown color pigment producer, and 2. Non-pigment producer. Pustules are loosely arranged into flat spreading blemishes. Diffused conidia, appearing powdery due to dense conidiation, rapidly turning yellowish green to dark green. The reverse of the plate seemed colorless to dark brown. Conidiophores were irregularly branched and formed on the aerial hyphae and had constricted base and verticillate branching (Fig. 9). Phialides were short and ampulliform and arranged in whorls having swollen centers with slender peaks. The conidial shape was globose to obovoid green and smooth-walled.

### *T. brevicompactum*

**Figure 10:**
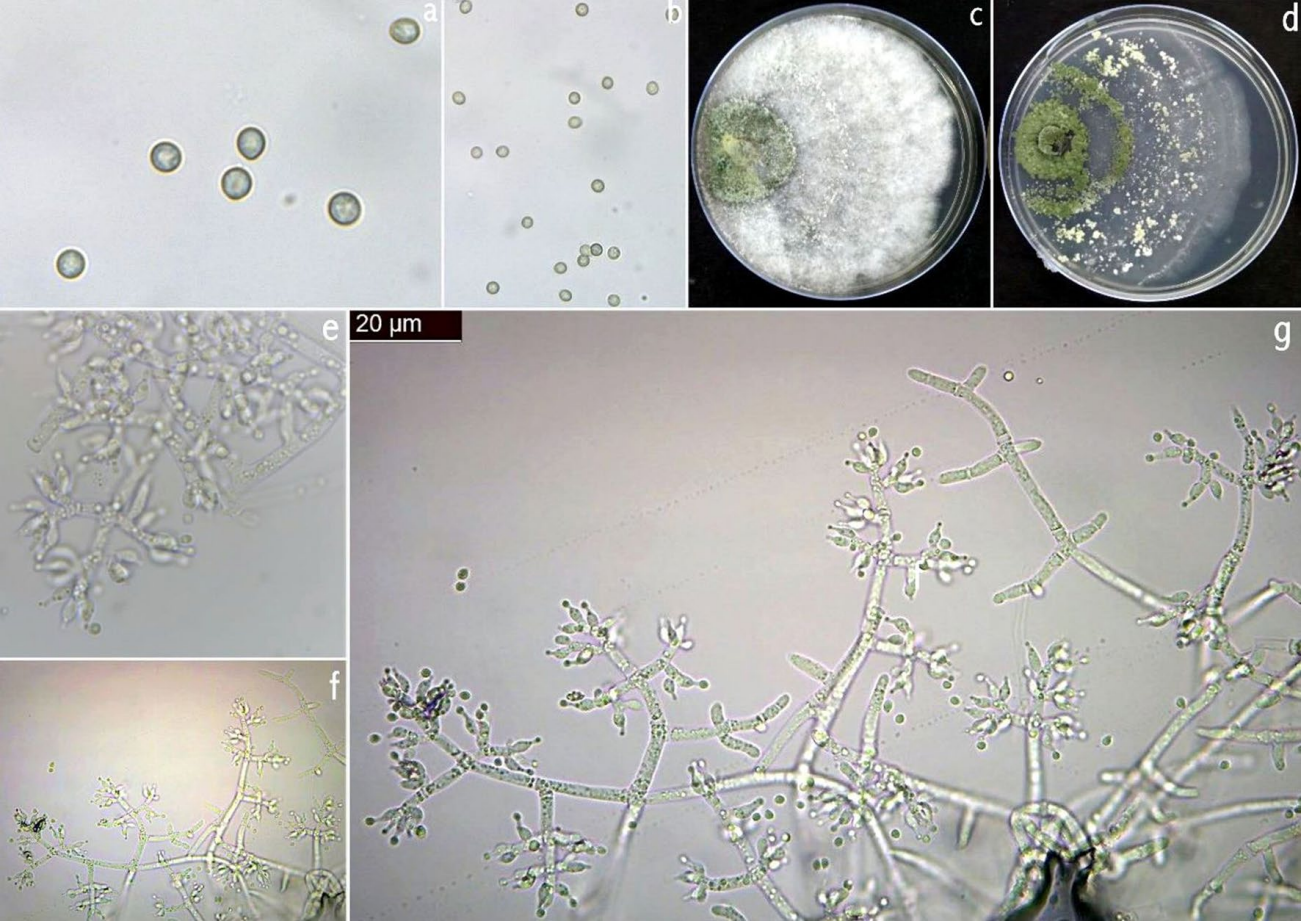
*Trichoderma brevicompactum*. (**a**, **b**) Conidia (PDA 2d); (**c**, **d**) Cultures at 27 °C, 7d (c. on PDA; d. on CMA); (**e**–**g**) Conidiophores and phialides (PDA 3d).

The colony of *T. brevicompactum* was characterized by densely aggregated conidiophores in coalescent pustules, which become greenish olivaceous to glaucous blue-green or grey olivaceous. On PDA, *T. brevicompactum* was fast growing, exceeding 75 mm on five days at 27 ± 1 °C. On CMA, the growth was a little slower and diffused, dispersed, and at the same time, formed concentric rings. The color of mature conidia in CMA was light green. In CZA, the colony growth was slower but had conspicuous concentric rings. In V8, no concentric rings were observed. Their conidial productions were restricted to the center of the colonies, diffused, and appeared to be yellowish green (Fig. 10). The conidiophores were hyaline, smooth-walled, pyramidally verticillately branched in the Pachybasium-type pattern. Conspicuous short sterile appendages are visible in young conidiogenous pustules but inconspicuous or absent in older cultures. Phialides were in whorls of 2–5, mostly broadly ampulliform with a short slender neck, solitary terminal with compact branches giving a compressed appearance to the conidiogenous structures. Conidia were subglobose or short ellipsoidal in some strains, mostly 2.0–3.0 mm in diameter, with a minimally protruding basal hilum, smooth-walled, appearing pale grey-green microscopically.

### *T. erinaceum*

**Figure 11:**
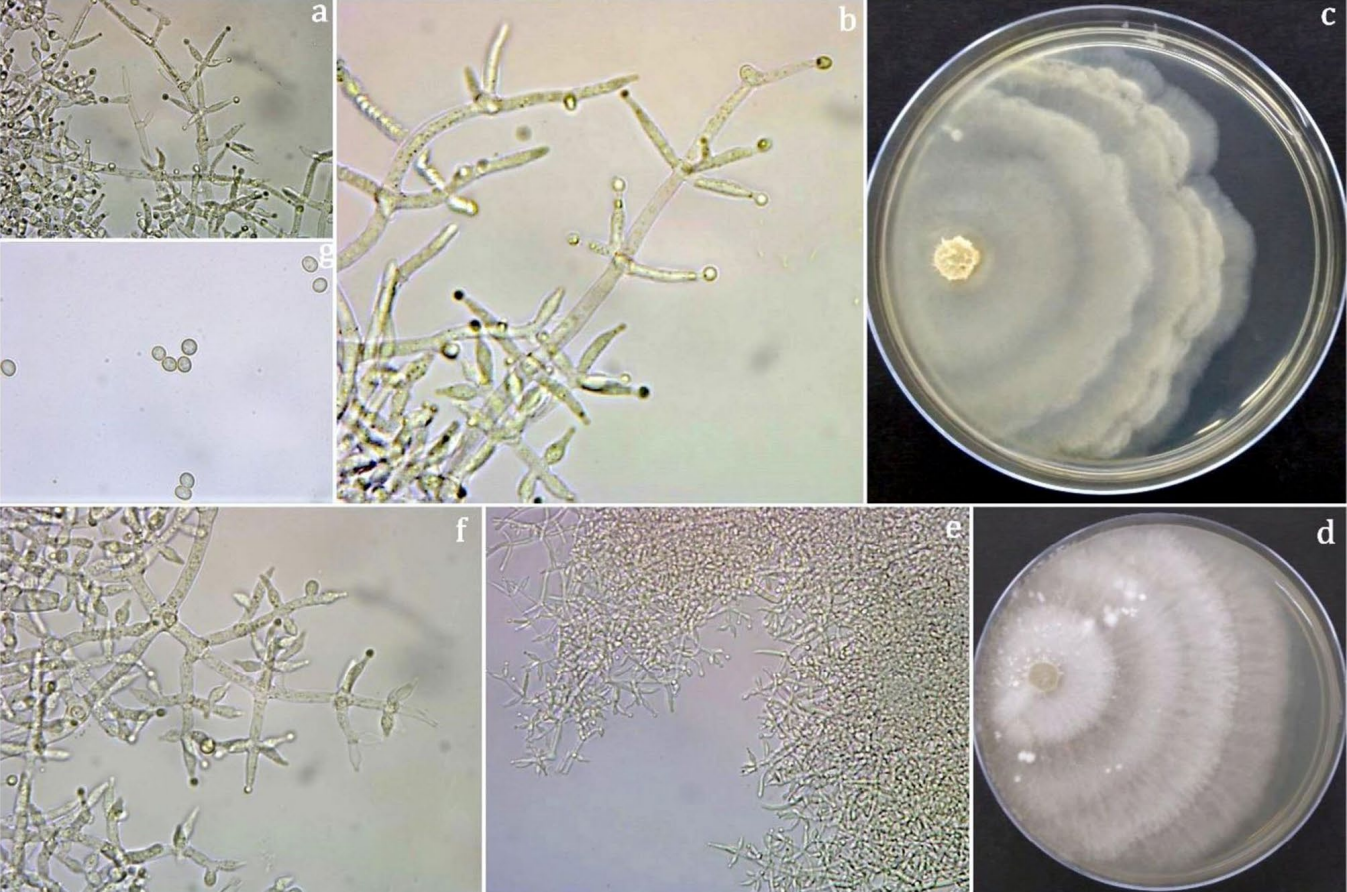
*Trichoderma erinaceum*. (**a**, **b**, **e**, **f**) Conidiophores and phialides (PDA, 3 d); **(c**, **d)** Cultures at 27 °C, 8d (c. on PDA; d. on CZA); **(g)** Conidia (PDA 4d).

Colony on PDA medium was moderately slow growing covering 25–30 mm in 72 h; mycelium covers plate on the 9th day after inoculation at 27 °C. Culture grows slowly, forming cottony white growth with concentric rings and turning green. Conidiophores were erect and arose at a right angle or less concerning the main branch. The phialides form a whorl of 2–3, having almost cylindrical to little swollen in the middle (6.0 to 8.0 µm long), conidia ellipsoidal to broadly ellipsoidal with a smooth margin (Fig. 11).

### *T. longibrachiatum*

**Figure 12:**
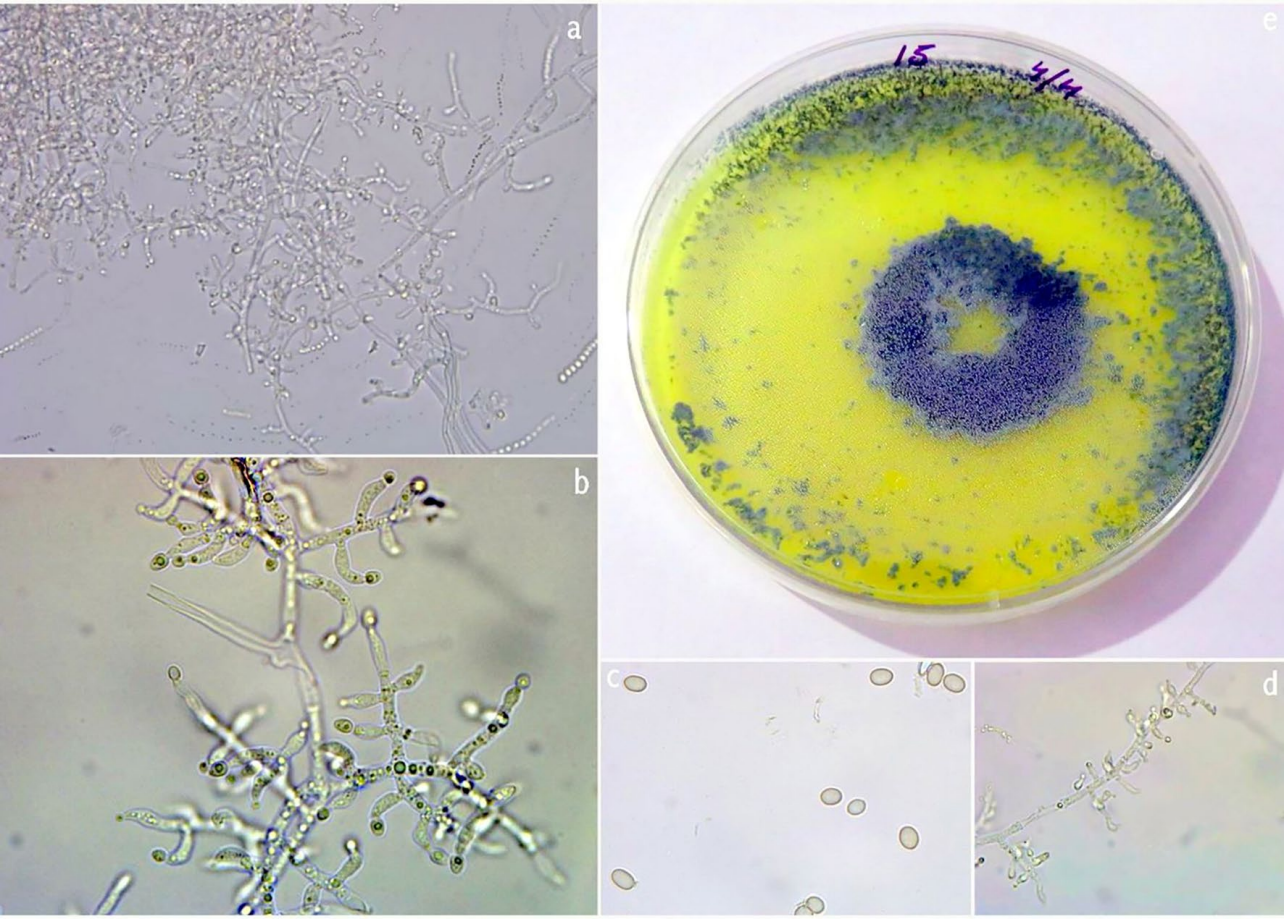
*Trichoderma longibrachiatum*. **(a, b, d)** Conidiophores and phialides (II); **(c)** Conidia (PDA 3d); **(e)** Culture at 27 °C on PDA.

Colonies on PDA grow well and rapidly change from white to green in the frequent occurrence of concentric ring-like zones. The culture produces lemon yellow pigment with this mycelial colony. Culture grows slowly; conidiophores form scanty aerial mycelium, cottony blue-green pustules. Conidiophores with a central axis on which phialides arise singly over several levels below the tip. Spur-like phialides occur as an outgrowth of the basal cell at the septum. Lageniform phialides are often cylindrical with a hook at the apex. Conidia are smooth, thick-walled, typically oblong, ellipsoidal, and smooth (Fig. 12).

### *T. asperellum*

**Figure 13:**
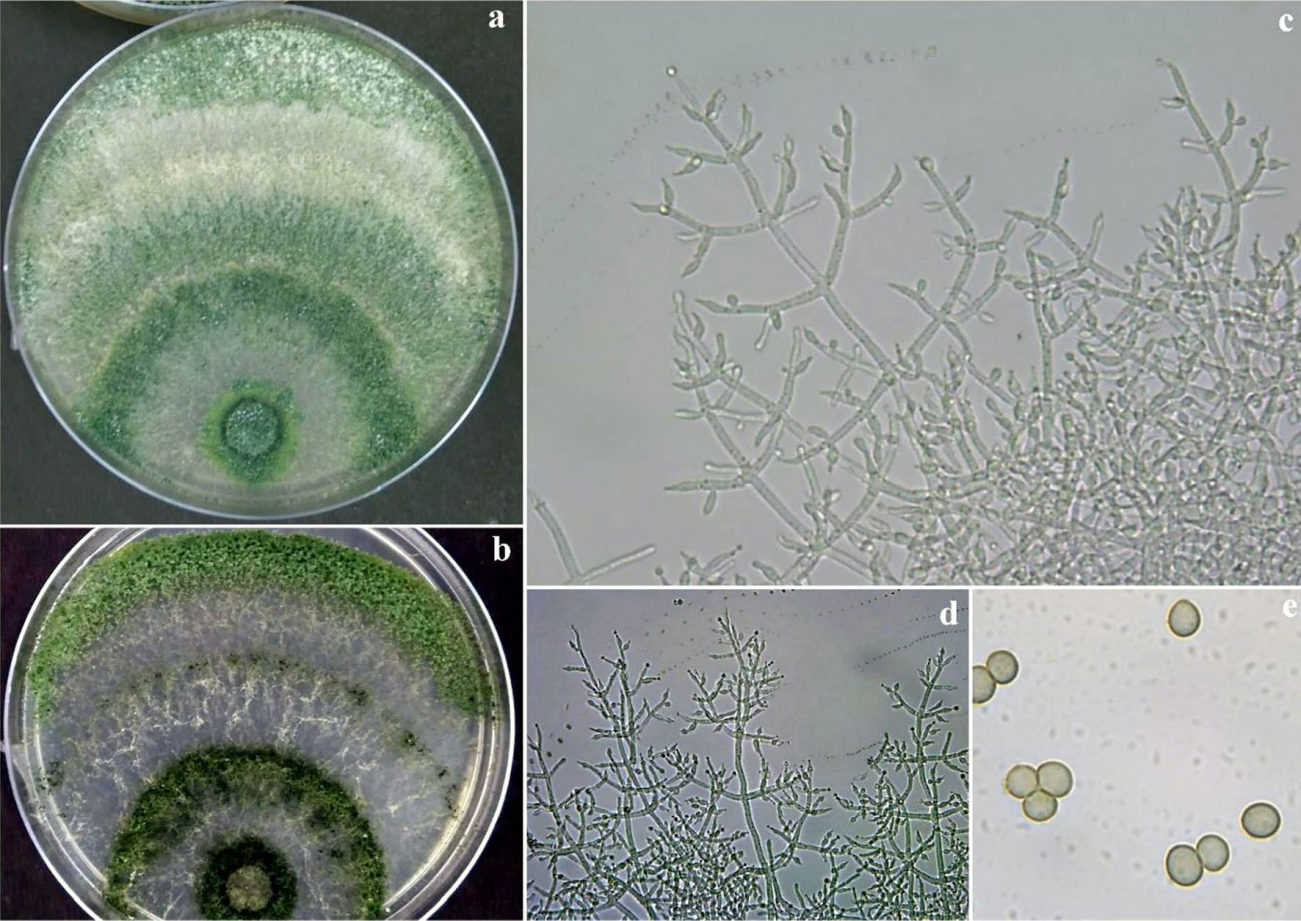
*Trichoderma asperellum*. **(a, b)** Cultures at 27 °C, 7d (a. on PDA; b. on CMA); **(c, d)** Conidiophores and phialides (PDA, 2d); **(e).** Conidia (PDA, 2d).

Colony on PDA medium was fast growing, covering 45–50 mm in 72 h, mycelium covers plate on 6th day after inoculation at 27 °C. The colony was dark green, and the mycelium color was white at the bottom. Mycelial morphology was coarse and formed concentric rings of dense conidial production, with conidia towards the center, dark green spores, and aerial mycelium lacking. Fertile, long, regularly branched conidiophores with more or less uniformly paired lateral branches and the most extended branch occurring at the distal end from the tip. Phialides form at the tip of branches in verticillate whorls. Phialides are long and slightly wider in the middle than at the base. Conidia are globose to slightly ovoidal (Fig. 13).

### *T. atroviride*

**Figure 14:**
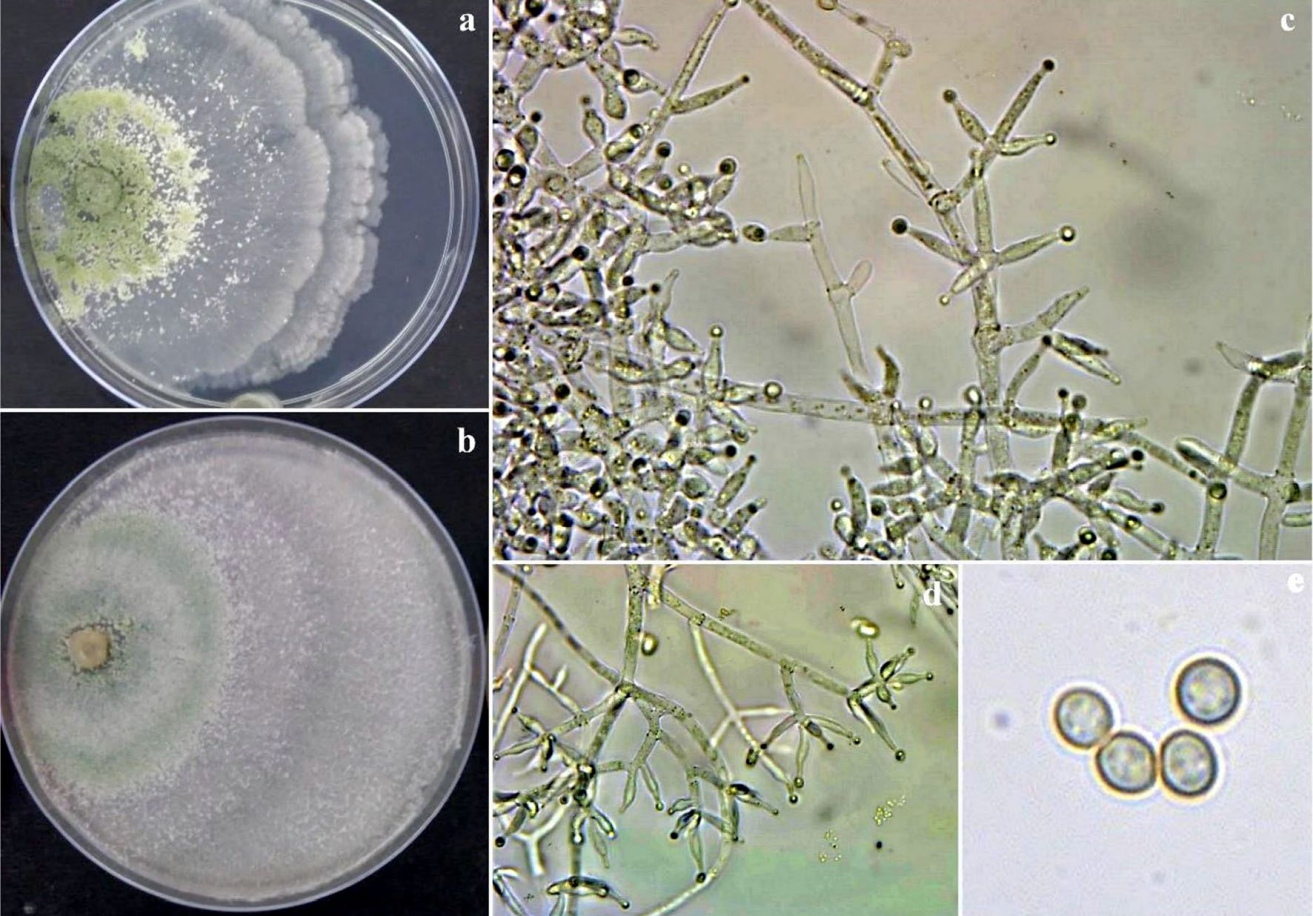
*Trichoderma atroviride*. **(a, b).** Cultures at 27 °C, 7d (a. on CMA; b on PDA); **(c, d)** Conidiophores and phialides (PDA 2d); **(e).** Conidia (PDA 2 d).

The colony character of the *T. atroviride* appeared as a tuft of dark greenish conidial mass having mature conidia in a central disk about 1.5 cm diameter and two pronounced, continuous white fluorescent concentric rings alternating with rings of sterile, felty white mycelium producing a single, terminal phialide (Fig. 14). Fertile branches arising at right angles from the base of setae, branches proximal to the tip of the seta typically comprising one or a few cells, terminating in a single phialide or a terminal whorl of 3–5 phialides; fertile branches produced lower, longer, and re-branching, each branch is having a whorl of phialides and solitary phialides arising directly from the branch axis. Conidia were subglobose to ovoidal.

### In vitro inhibition of *Sclerotium rolfsii*, *Rhizoctonia solani*, and *Fusarium verticillioides*

#### Antifungal activity of *Trichoderma* strains against *S. rolfsii*

**Figure 15:**
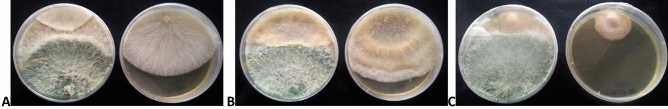
Confrontation assay of *Trichoderma* spp. Against (**A**) *Sclerotium rolfsii*; (**B**) *R. solani* and (**C**) *F. verticillioides.*

**Figure 16:**
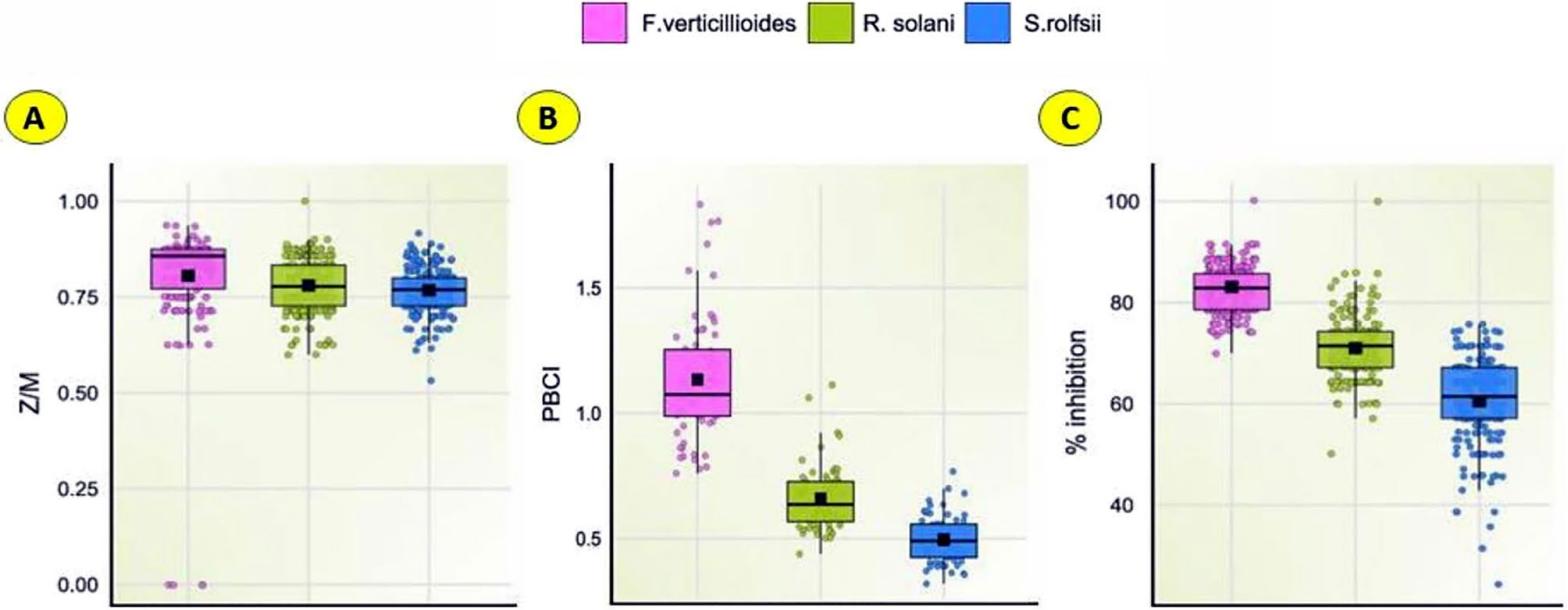
Represents the in vitro inhibition of *Fusarium verticillioides, Rhizoctonia solani,* and *Sclerotium rolfsii* by 60 *Trichoderma* strains through Box and Whisker Plots for the (**A**) pathogen resistance index (Z/M), (**B**) Pakdaman’s biological control indices (PBCIs) and (**C**) %inhibition.

**Figure 17:**
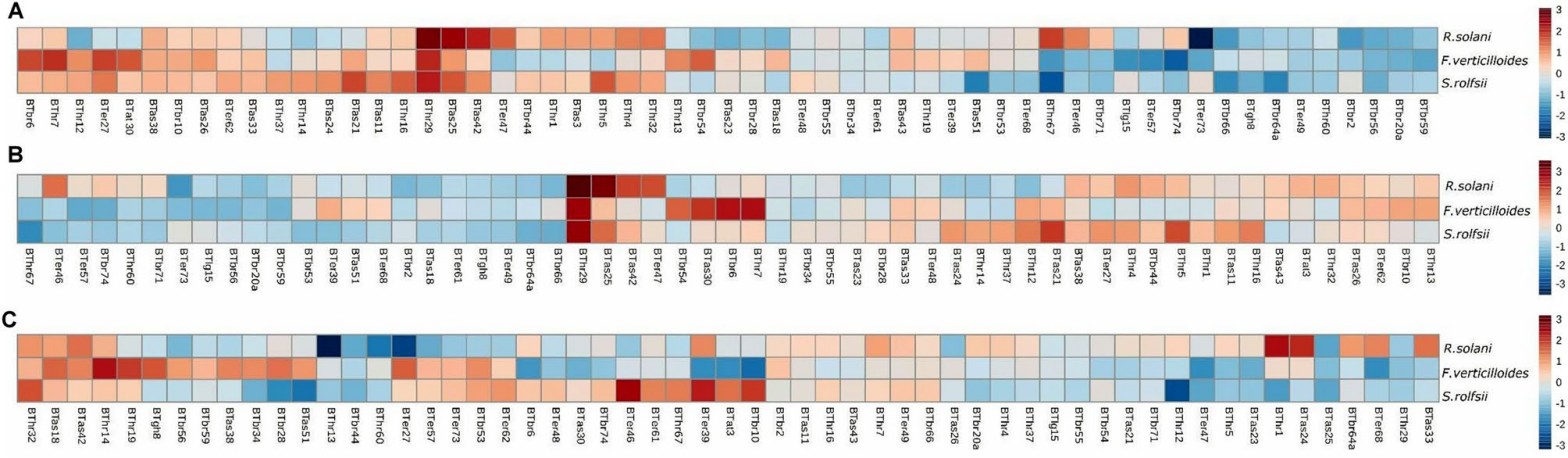
Heat map representing mean (**A**) Resistance index (Z/M), (**B**) mean PBCI value, and (**C**) mean % inhibition of *Rhizoctonia solani, Fusarium verticillioides,* and *Sclerotium rolfsii* by 66 *Trichoderma* strains. The data scaled between + 3 and − 3.

The results of dual culture indicated that all the isolates of *Trichoderma* spp. significantly (*p* ≤ 0.05) inhibited the growth of *S. rolfsii* from 44.8% (*T. afroharzianum* BThr67) to 74.8% (*T. afroharzianum* BThr29) on 6th day, restricting the pathogen almost completely on plates as compared to control consisting of *S. rolfsii* growing alone (Fig. 15A). Among all the *Trichoderma* isolates, the action exerted by *T. afroharzianum* isolates was relatively more potent (*P* ≤ 0.05) than that of other species of *Trichoderma* (BThr5 71.4%, BThr16 71.0%, BThr21 72.4%, BThr13 59.0%, BThr19 59.0%, BThr32 66.2% BThr35 62.4%, BThr37 67.1%) (Supplementary table 3). *T. asperellum* also showed at par efficacy to control *S. rolfsii* in confrontation assay. The range of *S. rolfsii* inhibition by *T. asperellum* was between 51.0% (*T. asperelloides* BTas51) to 72.4% (*T. asperellum* BTas21) (Supplementary table 3). Box plot model (Fig. 16C) depicts all data points of 60 *Trichoderma* strains causing % inhibition of *S.rolfsii.* The R-value (Z/M) indicated the radial growth of the pathogen in the presence of *Trichoderma* strains emphasizing the restricting potential of *Trichoderma* isolates. Lower the R value, higher the restricting potential. In the present study, the minimum radial growth with maximum restricting potential of *S. rolfsii* was 0.67 ± 0.02 showed by *T. camerunense* BThr12 and *T. brevicompactum* BTbr52. Other *Trichoderma* strains with lower R-value includes *T. brevicompactum* BTbr28 (0.70 ± 0.13), *T. brevicompactum* BTbr44 (0.70 ± 0.09), and *T. afroharzianum* BThr1 (0.71 ± 0.09). The Box plot model (Fig. 16A) indicates that Z/M values for all 60 isolates are below 1, a trend which was also indicated in the heat map (Fig. 17). Thus, all those isolates showed the good restricting potential of *Trichoderma* strains to prevent radial growth of the pathogen in plate culture and therefore found to be more aggressive towards the pathogen. However, the *Trichoderma* strains BThr29 with the maximum in vitro inhibition of *S. rolfsii* has R-value of 0.74 ± 0.02 (Supplementary table 3^3^). Pakdaman’s biological control index (PBCI) of BThr 29 was recorded to be the highest at 0.77 which clearly indicates the highest antagonistic potential of the strain against *S. rolfsii*. Other strains BThr 5 (0.68) and BTas25 (0.65) also showed their antagonistic potential against *S. rolfsii.*

### Antagonistic activity of *Trichoderma* spp. against *Rhizoctonia solani*

Dual culture assay of *Trichoderma* strains significantly inhibited the growth of *Rhizoctonia solani* from 61.9 ± 5.95% (*T. erinaceum* BTer73) to 84.8 ± 1.65% (*T*^*.*^* afroharzianum* BThr29) on 6th day after incubation (Fig. 15B). Heat map (Fig. 17) also shows the highest value near 3 to depict the intensity of inhibition of *R. solani.* The R-values of *T. afroharzianum* BThr13 and *T. erinaceum* BTer27 were very low 0.64 ± 0.04 and 0.67 ± 0.07, respectively (Supplementary table 4), indicating that there was less resistance from *R. solani* to the radial growth of *Trichoderma* strains which shows their antagonistic potential. Pakdaman’s biological control indices (PBCIs) of *Trichoderma* strains displayed in the Box plot model (Fig. 16B) and Heat map (Fig. 17) unambiguously indicated that among tested *T. afroharzianum* strains, the BThr29 has the highest PBCI value of 1.11 ± 0.12 followed by *T. asperellum* BTas25 (1.06 ± 0.24) which indicates its higher biological potential than other isolates against *R. solani.*

### Antagonistic activity of *Trichoderma* spp. against *Fusarium verticillioides*

Notable differences were observed for the radial growth of *F. verticillioides* in the presence of *T. afroharzianum* isolates. *T. afroharzianum* BThr29 caused maximum inhibition (89.5%) of *F. verticillioides* under in vitro conditions followed by other *Trichoderma* strains (BThr7, BTas30) (Fig. 15C). More inhibition of *F. verticillioides* by 60 *Trichoderma* isolates in comparison to other pathogens is clearly depicted by the median line in the Box plot model which is above 80% (Fig. 16C). The parameter of R-value (calculated as the ratio of Z/M) did not exhibit significant differences in resistance of *F. verticillioides* against *T. afroharzianum* isolates. However, Box plot model proved that all 60 isolates showed resistance lower than one (Fig. 16A), which suggested that growth media (PDA) facilitates more growth of *Trichoderma* isolates in the presence of *F. verticillioides*. BThr29, BTas23, BThr30, BTbr10, and BTer39 resulted in lower R values 0.78 ± 0.10, 0.77 ± 0.06, 0.77 ± 0.03, 0.73 ± 0.12 and 0.75 ± 0.13 respectively (Supplementary table 5) showing maximum resistance to the growth of *F. verticillioides*.

Pakdaman’s biological control indices (PBCIs) statistically differed highly, and it showed that BThr29, which has the highest PBCI value of 1.83 ± 0.58 was more effective than other *Trichoderma* strains in the biological control of *F. verticillioides* (Supplementary table 5)*.* Box plot model representing that median of PBCI value for all 60 *Trichoderma* isolates is above 1 (Fig. 16B) which showed that *Trichoderma* isolates outgrowth over slow-growing *F. verticillioides.*

### In vivo evaluation of the efficacy of potent *Trichoderma* strains to control damping off caused by *Sclerotium rolfsii* and *Rhizoctonia solani* in pot culture experiment

**Figure 18:**
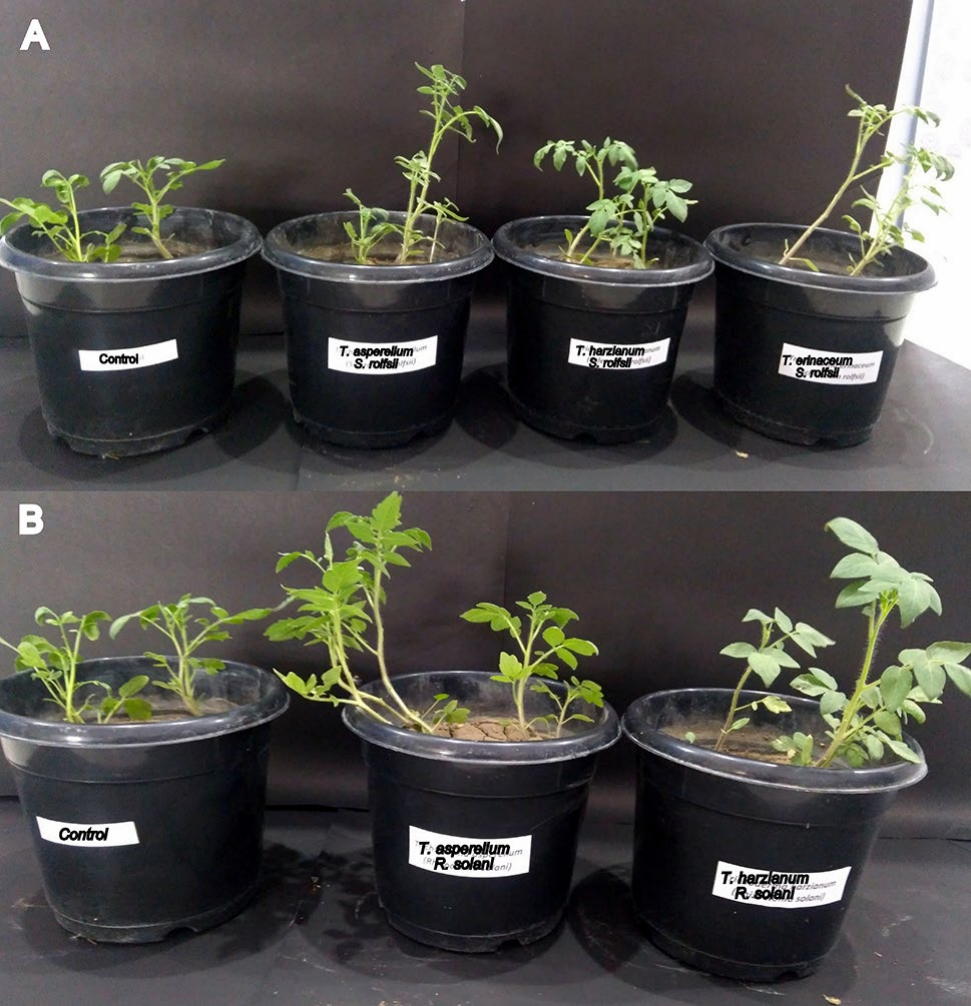
In vivo assay to assess potentiality of the effective *Trichoderma* strains in pot culture experiment. Stem length was measured 30 days post inoculation with *Trichoderma* strains. (**A**) Efficacy of *T. afroharzianum* BThr29, *T. asperellum* BTas25 and *T. erinaceum* BTer43 against *Sclerotium rolfsii* challenge inoculated in pots having tomato plants; (**B**) Efficacy of *T. afroharzianum* BThr29 and *T. asperellum* BTas25 against *Rhizoctonia solani* challenge inoculated in pots having tomato plants.

The most potential *Trichoderma* strains (BThr29, BTas25 and BTer43) obtained in the in vitro experiment were tested in pot culture experimenst (Fig. 18). Data in Table 1 showed the effect of potential strains on germination %, disease incidence, and plant growth promotion when inoculated with pathogens *S. rolfsii* and *R. solani*. The effects of *Trichoderma* treatments were significant when compared with the respective controls. In all the treatments in Table 1, the *Trichoderma* strains increased seed germination significantly (84.5–93.6%) compared to control treatments (66.3–67.1%). The *Trichoderma* treatments also reduced disease incidence in the tomato plants (5.2–11.2%) compared to 18.5–21.7% in control treatments. The beneficial effect of *Trichoderma* treatments also extended to plant growth promotion as measured by shoot length and weight. *T. asperellum* strain was slightly better than the other two strains for seed germination. On the other hand, *T. afroharzianum* strain was slightly better in controlling diseases caused by *S.* *rolfsii* and *R. solani. *The two strains of* T. asperellum *and* T. erinaceum *performed slightly better than* T. afroharzianum* strain in growth promotion in the tomato plants*.*

**Table 1:** Efficacy of *Trichoderma* isolates on damping off caused by *Sclerotium rolfsii*, *Rhizoctonia solani,* and plant growth promotion activities in tomato plant in pot culture experiment.

Trichoderma strains	*Sclerotium rolfsii*					*Rhizoctonia solani*				
	Germination(%)	Disease incidence (%)	% ROC	Shoot length (cm)	Shoot fresh weight (g)	Germination (%)	Disease incidence (%)	% ROC	Shoot length (cm)	Shoot fresh weight (g)
*T. afroharzianum* BThr29	84.5 (66.7)^a^	8.4 (16.8)^c^	61.2	13.8 ± 1.16^b^	2.14 ± 0.24^b^	89.8 (71.4)^b^	5.2 (13.1)^d^	72.0	13.4 ± 0.83^b^	2.34 ± 0.19^b^
*T. erinaceum* BTer43	85.7 (67.8)^a^	11.2 (19.5)^b^	48.6	17.9 ± 2.01^a^	3.36 ± 0.56^a^	87.5 (69.3)^b^	9.6 (18.0)^b^	48.4	16.2 ± 1.39^a^	3.18 ± 0.67^a^
*T. asperellum* BTas25	86.9 (68.8)^a^	10.7 (19.04)^b^	51.0	16.9 ± 1.27^a^	3.07 ± 0.48^a^	93.6 (75.8)^a^	8.0 (16.4)^c^	56.8	17.1 ± 0.81^a^	2.97 ± 0.62^ab^
Control	67.1 (55.0)^b^	21.7 (27.7)^a^	-	9.1 ± 0.99^c^	1.48 ± 0.29^c^	66.3 (54.5)^c^	18.5 (25.5)^a^	-	10.0 ± 0.74^c^	1.41 ± 0.4^c^
CV CD (P = 0.05)	6.85 2.45	6.63 1.39		9.8 2.18	8.14 0.64	4.22 4.41	4.24 1.19		6.89 1.51	12.3 0.78

*Data presented in mean of four replications; ROC = Reduction Over Control; # values in parenthesis are arc sine transformed values; Means in parentheses and ± standard error in the colums followed by the same letter do not differ significantly according to Duncan’s Multiple Range Test (DMRT).

## Discussion

Correct species identification is essential in selecting and validating microbial biocontrol agents^6^ thus, it is pertinent to assess intraspecific variation to understand the species diversity available in the crop rhizosphere. The phylogenetic species concept has added benefits over traditional morphology-based identification. In the present study, the *Trichoderma* species isolated from the crop rhizosphere of southern Rajasthan were identified based on morphology and *tef-1α* sequence analysis to delineate the species and phylogenetic analysis to report the level of *Trichoderma* biodiversity in agricultural soil. Of the 11 species identified, 4 dominant species *T. brevicompactum* (18), *T. afroharzianum* (12)*, T. erinaceum* (11), and *T. asperellum* (10) showed ubiquitous distribution in studied districts of southern Rajasthan. The higher abundance of these four species may be due to high genetic diversity which shows their potential to adapt to varied environments. Recently, the biodiversity of *Trichoderma* has been widely studied in different parts of the world. But those studies were designed to discover newer species from non-agricultural soils^28–31^. However, there are very few studies that focus on *Trichoderma* diversity in agricultural soils internationally and in India^32^ in China^33^, in Ethiopia^18^, in Manipur^20^, in Uttarakhand), and little effort was made to study the actual distribution and association of *Trichoderma* species in various crop rhizosphere. The biodiversity evaluation is important for agricultural soil to find the most potential isolate of *Trichoderma* species which can be further developed as a bioformulation to manage various soilborne pathogens. In this study 60 *Trichoderma* isolates were isolated from seven districts of southern Rajasthan covering 16 crops in the rhizosphere. These seven districts are representative of two agroclimatic zones namely, Zone IVA-Sub-humid southern plains and IVB-Humid southern plains. Agriculturally fertile land of the southern humid zone (Zone IVB) has 72% isolated strains as compared to the remaining 28% from Sub-humid southern plains (Zone IVA). Agro-ecological condition of collection sites in zone IV B has 900 to 1000 mm average rainfall with a thick density of forests, shrubs, and cropping intensity of more than 100%. Zone IV A sub-humid southern plan has 700–900 mm average rainfall with relatively thinner vegetation^34^. This suggests that the isolation frequency of *Trichoderma* isolates largely depends on a better ecological environment (minimum–maximum temperature, relative humidity, rainfall) and higher cropping intensity. Researchers^28,35–37^ also support that the *Trichoderma* diversity is more in areas with higher vegetation and fertile and as compared to the region with poor ecological environment. *T. brevicompactum*, *T. afroharzianum,* and *T. asperellum* isolates were mainly restricted to the irrigated regions of southern Rajasthan including Banswara, Dungarpur Pratapgarh, and Chittorgarh. Previously, it was reported by Maina et al.^38^ that *T. brevicompactum* was restricted to the irrigated regions, in addition, our report suggests that not only *T. brevicompactum* but *T. afroharzianum,* and *T. asperellum* also predominance in irrigated regions. *Trichoderma brevicompactum* was described by^39^ and later revisited by Degenkolb et al.^40^ and was shown to be in a new lineage with few other closely related species. They also reported that *T. brevicompactum* has no close relationship to species used in biological applications. *T. ghanense* was isolated from the undisturbed forest area of Pratapgarh during the summer season. *T. erinaceum* is isolated from areas with lesser humidity and dry regions of Pratapgarh, Udaipur, Chittorgarh, Rajsamand, and Sirohi districts. Maina et al.^38^ also reported the prevalence of *T. ghanense,* and *T. erinaceum* in hot areas.

In this study, we observed *T. brevicompactum* is the most commonly occurring species as compared to many previous reports which suggest *T. harzianum* is a commonly reported species^28,41^. *T. afroharzianum* is the next most commonly occurring species in the present study. We found *T. afroharzianum, T. inhamatum,* and *T. camerunense* encompassing Harzianum clade as has been reported by Chaverri et al.^6^ that *T. harzianum* is a species complex. Next dominant species including 12 strains reported in our study was *T. afroharzianum*. This species has worldwide distribution and well-known in biocontrol applications. In fact, the famous commercial isolate T-22 was found to be *T. afroharzianum* (KP008850)^6^. *T. harzianum* was split into more than 14 species with *T. harzianum* representing only one of those 15 species^6^. There are two other reference sequences in the *T. harzianum* clade which have been used in biocontrol but deposited in GenBank under the old name (*T. harzianum*). The newly assigned *T. harzianum* has rare distribution in Europe and North America. Another closely related strain in Harzianum clad was *T. lentiforme* BThr67. In fact, BThr67 had an identical sequence to *T. lentiforme* from Mexico. *T. lentiforme* is known as South American species and mostly as endophyte^6^. To our knowledge this is the first report of the species outside the South American continent. *T. erinaceum* and *T. asperellum* were the next dominant species in southern Rajasthan. Samuels et al.^7^ described *T. asperelloides* as new species out of *T. asperellum*. Our study indicated 10:3 ratio among *T. asperellum* and *T. asperelloides* within the strains isolated in India indicating the dominance of *T. aseprellum* over *T. asperelloides* in the isolation region. Both of these species have been used in biocontrol applications^42–44^. Longibrachiatum section consisting of two strains, *T. longibrachiatum* BTlg15 and *T. ghanense* BTgh8 known producers of cellulose hydrolyzing enzymes (particularly *T. reesei*^45,46^), as cause of opportunistic infections of man and animals^47,48^.

The identities of 60 isolates were established by morphological and PCR amplification of the rDNA *tef-1α* primer sequence. Many researchers demonstrated the effectiveness of *tef-1α* marker delineating variations to discriminate among different *Trichoderma* species^30,49,50^. *Trichoderma* species included among the 60 identified isolates were *Trichoderma afroharzianum*, *T. asperellum*, *T. asperelloides*, *T. inhamatum*, *T. camerunense*, *T. erinaceum*, *T. atroviride*, *T. ghanense*, *T. lentiforme, T. longibrachiatum*, and *T. brevicompactum*. *T. atroviride* is known to be cosmopolitan in nature but more common in northern temperate regions^51^. Moreover, among strains identified in this study, *T. afroharzianum, T. asperellum, T. asprelloides, T. atroviride, T. brevicompactum,* and *T. erinaceum* are well-known as biocontrol species^52,53^.

However, these species can be distinguished from other species by having species-specific features, viz., different colony growth characteristics, colony color, shape, size, and arrangements of phialides. In our study, these species were grown on different media to study colony morphology. The *T. afroharzianum, T. asperellum, T. ghanense, T. erinaceum, T. longibrachiatum,* and *T. atroviride, T. hamatum* formed distinguished concentric rings of conidia on different media. The rings were thicker and denser on PDA and CMA media under alternating light and dark conditions of photoperiod. *T. brevicompactum* under these photoperiod conditions showed broken rings while *T. erinaceum* and *T. atroviride* showed wavy conidial rings. Steyaert et al.^54^ reported that 15 min burst of blue light could produce clearly defined concentric rings. The authors also suggested that in addition to primary N present in media, the supply of secondary N produced distinguished rings in PDA media. The formation of concentric rings of conidia is the response of alternating light and dark photoperiod, and in single light exposure, a single ring of profuse conidiation will be observed at the colony margin^55^.

In the present study, it was observed that *Trichoderma* species communities in various crop rhizosphere varied significantly. The crop rhizosphere of soybean represents the maximum number of 10 strains covering four species including *T. erinaceum*, *T. brevicompactum, T. afroharzianum, T. asperelloides, T. inhamatum*, and *T. lentiforme*. Maize crop rhizosphere represents nine strains covering three species seven strains of *Trichoderma*, including *T. afroharzianum*, *T. erinaceum, T. asperelloides*, and *T. brevicompactum.* The paddy crop represents only one species *T. afroharzianum*. The cucumber crop rhizosphere represents four species, namely *T. afroharzianum, T. brevicompactum, T. asperellum,* and *T. longibrachiatum*. Crop rhizosphere of Cluster bean represents only *T. atroviride*, sugarcane represents only *T. brevicompactum* and okra crop represents only *T. asperellum*. This variation may be due to the composition of a complex mixture of bioactive root exudates to modify the composition of the rhizospheric microbiome^56^. Lombardi et al.^57^ demonstrated that different biotic and abiotic stress elicit root exudates from tomato crops to increase the chemo-attractive effect on *T. harzianum*. Thus, it is considered that root exudation in different crop rhizosphere is responsible for variation in *Trichoderma* communities. Our study showed that the rhizosphere of soybean and maize crops harbor more isolates and species than other hosts. That may not only be due to the root exudates of these crops but also due to the existence of phenolic acids in their rhizosphere having synergistic antimicrobial activity by modulating microbial proliferation^58^.

To study the morphological and cultural variability of isolates, it is pertinent to grow them in different mediums. The growth medium has a significant effect on the growth rate and morphology of fungi. The respective media (i.e., PDA, CMA, TJA) were chosen in this study partly because of their widespread adoption in similar studies by other workers^59,60^ and because of their inherent variability in composition. It was observed that *Trichoderma* isolates grew poorly on TJA media but showed luxuriant growth on PDA and CMA. The growth performance of *T. harzianum* was studied by Jahan et al.^59^, and the highest linear growth, fresh weight, and dry weight were found in potato dextrose agar followed by carrot agar and corn meal agar (CMA). There are several reports which support our observation that PDA is the most suitable media for the growth of *Trichoderma*^60,61^. Our results showed that minimal media TJA did not sustain the luxuriant growth of different *Trichoderma* species; this was also supported by the work of Harman et al.^23^ who reported high conidial production of *T. harzianum* but with an overall low yield.

A confrontation assay of *Trichoderma* strains against *S. rolfsii, R. solani,* and *F. verticillioides* was based on the direct radial growth of the pathogen in the presence of *Trichoderma* strains, and in the assay, the parameters of pathogen resistance against *Trichoderma* strains were evaluated. R value (Z/M) of the confrontation assay against all three pathogens was less than 1, which clearly indicates that none of the pathogens resisted but permitted the growth of *Trichoderma* strains^62^. That implies that the strains with lower R values are more potent. Nevertheless, the strain *T. afroharzianum* BThr29 caused maximum inhibition of *S. rolfsii* (74.8%), *R.solani* (84.8%), and *F. verticillioides* (89.5%) with comparatively higher R values. This shows that R-value is not the only parameter to decide the potentiality of *Trichoderma* strains. The Pakdaman’s biological control indices (PBCIs) of *Trichoderma* strains evidently indicated that the tested *T. afroharzianum* BThr 29 has the highest PBCI values of 0.77 ± 0.02, 1.11 ± 0.12, and 1.83 ± 0.58 respectively against *S. rolfsii, R. solani,* and *F. verticillioides*, indicating that the BThr29 strain of *Trichoderma* has more biological control potential than other strains against *S. rolfsii.* Based on these PBCI values obtained in the study *T. afroharzianum* BThr29 is the most potential *Trichoderma* strain. A good amount of *Trichoderma* biodiversity in the crop rhizosphere of southern Rajasthan was identified in this study. Our study was based on isolates collected during the summer season. Some species sporulate or proliferate in some other seasons. Thus, seasonal variability must be considered to explore other seasons of crop rhizosphere to collect more information in future surveys of *Trichoderma* diversity in crop rhizosphere.

Potentiality of the effective *Trichoderma* strains were assessed by their efficacy evaluation in the in vivo pot culture experiments.Several studies have shown that *Trichoderma* prevented diseases caused by soil-borne pathogens such as *Fusarium* spp^63,64^, *Pythium* spp.^65^, *R. solani*^66,67^*,* and *Sclerotium rolfsii*^68,69^. In our previous study, we evaluated the efficacy of *Trichoderma* strains BThr29, BTer43 and BTas25 against *Fusarium* species complex causing post flowering stalk rot in maize. These *Trichoderma* strains not only reduced disease severity from 17.58 to 62.37% but also improved crop stand by reducing lodging from 42.1 to 71.1%^43^. In the present study, it was found that *T. afroharzianum* BThr29 not only effective in managing pathogen growth in in vitro experiments but also was found to reduce the disease incidence by *S. rolfsii* and *R. solani* when challenge-inoculated in pot culture experiments. This may be because *T. harzianum* has high chitinase- and β 1,3 glucanase-producing ability^70^. Though, *T. afroharzianum* BThr29 was found to be most effective in controlling diseases incidence, treatments with *T. asperellum* and *T. erinaceum* BTer43 were also observed to improve plant growth promotion. There are reports which suggest that *Trichoderma*, promotes plant growth and development through several mechanisms including stem and root growth, increased nutrient uptake, and expression of plant defense-related genes^71^. The biopriming of tomato plants with *T. erinaceum* induces profuse growth in plants by inducing stem and root growth^72^.

In conclusion, the findings of our study present detailed information on the diversity of *Trichoderma* species in the crop rhizosphere of southern Rajasthan, India. A total of 60 isolates of *Trichoderma* were identified up to species level based on *tef-1α* primer sequence analysis. *T. afroharzianum* (BThr29) showed high antagonistic potential against three soil-borne pathogens *S. rolfsii, R. solani*, and *F. verticillioides.* To the best of our knowledge, this study first time reports the occurrence of three cryptic species of *T. harzianum* species complex from India. They include *T. lentiforme, T. camerunense*, and *T. inhamatum*. The strains *T. afroharzianum* BThr29 and *T. asperellum* BTas25 were found to be efficient antagonists, while *T. asperellum* BTas25 and *T. erinaceum* BTer43 have good growth promotion potentials.

## Materials and methods

### Geography of sampling sites

*Trichoderma* isolates in this study were obtained from soils of cultivated lands and orchards in 7 districts (Banswara, Udaipur, Dungarpur, Pratapagarh, Rajsamand, Sirohi, Chittorgarh, Pali) of southern Rajasthan (Fig. 1). The sampling sites were located in two agro-climatic zones, viz. Zone IVa-Sub Humid Eastern Plains, and Aravalli Hills and Zone IVb-Humid Southern Plain. We collected 273 soil samples during March, April, and May of 2017. The geographical coordinates of the places from where samples were collected are depicted in Supplementary table 1, and the soil samples were collected from diverse crop rhizosphere of cereals, pulses, vegetables, fruit plants, and forest soil as depicted in Fig. 1. Each sample contained about 200 g of soil from a depth of approximately 20 cm. The samples were kept in clean polythene bags, transported to the laboratory, and stored at 4 °C until isolation.

### Isolation and storage of *Trichoderma* strains

Five-fold serial dilutions of soil samples were prepared in sterilized distilled water and 1 ml of diluted sample was poured on the surface of *Trichoderma* selective Medium (TSM) (MgSO_4_*7H_2_O 0.2 g + K_2_HPO_4_*3H_2_O 0.9 g + NH_4_NO_3_ 1.0 g + KCl 0.15 g + rose Bengal 0.15 g + glucose 3 g + agar 20 g liter dissolved in 1 L of sterile distilled water. Then added 1 ml of a solution containing FeSO_4_.7H_2_O 1 g, MnSO_4_.4H_2_O 0.65 g, and ZnSO_4_.7H_2_O 0.9 g. Autoclaved the TSM at 121 °C, 15psi for 15 min. Before pouring TSM 0.5 ml of Chloramphenicol @ 5 mg ml^−1^ was added as a bacterial inhibitor. Plates were incubated at 28 ± 2 °C in the dark, and on the second day, characteristic distinct colonies were spotted on the plates. After spotting the desired *Trichoderma* colonies, they were confirmed under a microscope (20 × and 40 ×) for the presence of phialides. Putative *Trichoderma* colonies were single spore purified by two rounds of sub-culturing on potato-dextrose agar (PDA) and kept in a refrigerator (4 °C) for further studies.

### Morphological characterizations

#### Growth rate

For morphological analysis, the growth of *Trichoderma* isolates was observed on grown on three different culture media. The composition of each for 1 L was: (1) Corn Meal Agar (CMA) media (Corn meal infusion 50 g + Dextrose 2 g + Agar 15 g), (2) Potato Dextrose Agar (PDA) media, and (3) Tomato juice agar (TJA) media (Tomato juice 100 ml + L-Asparagine 10 g + Yeast extract 2 g + calcium carbonate 2 g + glucose 2 g agar 20 g). Mycelial plugs (5 mm) from the margin of growing fungal colonies were cut and placed 1 cm away from the periphery of 9 cm agar plates. The culture plates were then incubated in the dark at 28 ± 2 °C. A duplicate plate for each isolate was prepared. The diameter of each colony in mm was measured every 24 h-interval until the agar plates were fully colonized. The cultural phenotypic characteristics were observed using digital camera (Canon DSLR- EOS 80D).

### Cultural characteristics

Morphological observations were based on fresh cultures grown on PDA for up to one week in an incubator at 28 ± 2 °C in the dark. Cultural descriptions are based on growth observations on PDA because of superior sporulation and more normal development of conidiophores. Characteristics of conidium-bearing structures and conidia were assessed from cultures grown on PDA. *Trichoderma* isolates were identified up to the genus level based on colony characters, growth, and structure of mycelium, conidiophores, phialides, and conidia as described by Kubicek and Harman^73^. The microscopic observations of 2–3 days from young culture were made from slides by mounting mycelia from the edges of actively growing culture on the sterile distilled water drop. Observations of the conidiophores and structure of phialides were made under a Leica DM LB microscope (Leica Microsystems, Wetzer, Germany), and an ES4 Leica microscope camera (Leica Microsystems, Wetzer, Germany) was used for microscopic photographs.

### Molecular characterization

DNA extraction, amplification, and sequencing-

*Trichoderma* mycelia were obtained from cultures on PDA plates grown at 28 ± 2 °C for 4–5 days. An approximately 5 mm^2^ agar plug with mycelium was cut and transferred to a 1.5 mL Eppendorf tube. The agar plug was ground with motor driven tissue grinder, and 600 µL CTAB (cetyl trimethyl ammonium bromide) buffer [Tris- HCL 100 mM, EDTA (Ethylene diamine tetra acetic acid) 20 mM, NaCl 1.4 M, CTAB 2% (w/v), β-mercaptoethanol 0.2% (v/v), pH 8.0] was added. The homogenate was incubated at 65 °C for 30 min. Subsequently, 600 µL chloroform-isoamyl alcohol (24:1) was added to the solution, mixed, and centrifuged (Eppendorf™ 5727R) for 10 min at 17,709×*g*. The upper aqueous layer was transferred to a new tube. 600 µL chloroform-isoamyl alcohol (24:1) was added, repeating the previous step. 300 µL isopropanol was added to the aqueous phase obtained from the previous step and incubated at − 20 °C for 30 min. Then, the mixture was centrifuged for 10 min at 12,000 rpm (16,128×*g*). The precipitate was washed with ice-cold 70% ethanol, dried at room temperature, re-suspended with 150µL double distilled water, and stored at − 20 °C.

For amplification of the *tef-1α* section, the forward primer tef1 F5′-CAT YGA GAA GTT CGA GAA GG and the reverse primer tef1 R5′-GGA RGT ACC AGT SAT CAT GTT^74^ were used. The PCR reaction was performed in a thermal cycler (Bio-Rad® Laboratories India Pvt Ltd, Gurugram) using a program with the following parameters: 5 min initial denaturation at 95 °C, followed by 35 cycles of 1 min denaturation at 94 °C, 45-s primer annealing at 54.3 °C, 30-s extension at 72 °C. The program was concluded with a final extension at 72 °C for 5 min. Aliquots of 2 µl of PCR reactions were analysed by electrophoresis in a 1% agarose gel, stained with ethidium bromide for visualization under the Gel Doc EZ system Bio-Rad® (Bio-Rad® Laboratories India Pvt Ltd, Gurugram). PCRs positive for products were purified using a QIAquick® PCR purification kit (Qiagen Inc., Chatsworth, CA, USA). Purified PCR products were sequenced in both directions using the primers used to generate them, on an Agile BioScience (Mumbai) sequencer.

### Phylogenetic analysis

The *tef-1α* sequences were assembled and edited using the software Sequencher from Gene Codes Corporation, Ann Arbor, MI USA (http://www.genecodes.com). The sequences were placed in one FASTA file with reference sequences obtained from GenBank based on homologies with our isolates and aligned all the sequences using the online tool (Guidance server) available at http://guidance.tau.ac.il/. The aligned file was used to generate phylogenetic trees using Maximum Likelihood criterion in MEGA X with the substitution model predetermined with MEGA X as well. Supports for the branches were assessed with 1000 bootstrap replicates. Phylogeny trees were also constructed using PAUP4 version 4a169 available online at http://phylosolutions.com/paup-test/. The tree was obtained using parsimony in heuristic search, with starting tree obtained via random stepwise addition and TBR as branch swapping algorithm plus Multrees in effect. The maximum likelihood tree had essentially identical topology to the parsimony tree and thus we presented the parsimony trees but the bootstrap values from Maximum likelihood tree are depicted on the branches of the presented trees.

### Antagonistic activity of *Trichoderma* strains against *Sclerotium rolfsii*, *Fusarium verticillioides*, and *Rhizoctonia solani*

The antagonistic potentials of *Trichoderma* strains against *S. rolfsii, R. solani,* and *F. verticillioides* were evaluated by following the procedure described by Comporta^75^. Mycelial discs of seven days old cultures of *Trichoderma* strains (60 no.) and fungal pathogens (*S. rolfsii, R. solani,* and *F. verticillioides*) from PDA grown at 27 ± 2 °C under dark conditions were placed at 1 cm away from the periphery at opposite ends on Petri dishes having PDA medium. Three replications were kept for each fungal-fungal interaction, and the biocontrol potential of each *Trichoderma* strain was studied against the three pathogens. *Trichoderma* strains and pathogens discs alone were kept on PDA in Petri plates as control. All these plates were incubated at 27 ± 2 °C in the dark, and the periodic growth of fungi was measured for 10 consecutive days. The inhibition was calculated at six days of incubation using the formula given by Mokhtar and Aid^76^.

$$\% {\text{ I}}\, = \,\left( {{1} - {\text{T}}/{\text{C}}} \right)\, \times \,{1}00$$

where, %I = Inhibition percentage of pathogen growth by *Trichoderma* strains; C = Radial growth in control (cm); T = Radial growth of test culture (cm).

Other biocontrol parameters were determined as suggested by Pakdaman et al.^62^ including (i) days required for two colonies to come in contact (I),(ii) days required for the BCA to fully grow over the pathogen colony (Z); (iii) days required for the BCA to fully grow over the plate (M); (iv) the radial growth distance (in cm) of pathogen colony between the point of inoculation and the marginal point of contact with the BCA growth zone (P); (v) the pathogen resistance index to the BCA was defined as the ratio of Z/M based on the periods required for the full growth of a fungal BCA in the presence (Z) and absence (M) of the pathogen^62^. Pakdaman biological control index (PBCI)^62^ combining temporal parameters and pathogen growth parameters was calculated following the formula given below:

$${\text{PBCI }}\left( {{\text{in cm}}^{{ - {1}}} } \right) = {\text{M }}\left( {\text{in days}} \right)/({\text{Z }}\left( {\text{in days}} \right){\text{ x P }}\left( {\text{in cm}} \right))$$

### In-vivo efficacies of potent *Trichoderma* strains

The potent *Trichoderma* strains *T. afroharzianum* BThr29, *T. asperellum* BTas25 and *T. erinaceum* BTer43 obtained in the in-vitro evaluation against *S. rolfsii* and *R. solani* were evaluated for their efficacies in the pot culture experiment. The experiment was designed with four replicates of the four treatments with randomised block design. The four treatments include seed treatment and soil drenching of *T. afroharzianum* BThr29, *T. asperellum* BTas25, and *T. erinaceum* BTer43 in challenge-inoculated pots with *S. rolfsii* and *R. solanii.* Pathogen inoculums were prepared on maize sand meal (MSM) media^77^. Fifteen days old MSM media with sufficient inoculums of the two pathogens were uniformly distributed (5 g/Kg of sterilized soil) in each pot. Spore suspensions (1 × 10^7^ cfu/ml) of freshly grown *Trichoderma* strains were inoculated through root irrigation. Non-inoculated pots were kept as controls. Twenty (20) surface sterilized tomato seeds per pot were treated with slurry of individual *Trichoderma* strains and were allowed to soak for 30 min. The treated seeds were placed in sterile 90 mm Petri plates and air dried in laminar flow bench overnight at room temperature and then used for sowing in the glass house study. Germination % was measured at 10 days after sowing. Thinning was done to maintain 5 seedlings per pot. Disease incidence was measured on the basis of infected seedlings out of a total number of seedlings. Each seedling was inoculated with 3 mL of individual Trichoderma strain suspension. *Trichoderma* treated seeds were evaluated for germination %, shoot length (in cm), shoot fresh weight (in g), and incidence of damping off of seedlings. Shoot length and shoot fresh weight were measured 30 days after sowing. The incidence of damping off in seedlings was expressed as a percentage of the total number of plants.

### Statistical analysis

The data collected were analysed by ANOVA technique using statistical package for social science (spss version 20.0). Experimental design was completely randomised design (CRD) for in vitro experiments and for pot culture experiment, the randomised block design (RBD) was used. The data of germination percentage, disease incidence were subjected to analysis of variance with arcsine transformation of percentage values and treatment means were separated by Duncan’s Multiple Range Test (DMRT) at a probability level 0.05, where treatment effects were significant. Box plots were created to depict the growth of *Trichoderma* spp. in growth media at different days after inoculation. The correlation study was performed using Jamovi version 1.2.27 at 5% level of significance.

### Supplementary Information

Supplementary Tables.

## Data Availability

Sequence data of identified *Trichoderma* strains used in the current study are available in NCBI archives https://ncbi.nlm.nih.gov/. The name of the repository and accession number can be found here; https://www.ncbi.nlm.nih.gov/genbank/, NBCI GenBank accession numbers are. OQ362340, OQ362310, OQ362336, OQ594958 OQ362342, OQ362304, OQ362337, OQ362349, OQ362302, OQ362312, OQ362351, OQ362339, OQ362344, OQ362348, OQ362338, OQ362320, OQ362350, OQ362295, OQ362319, OQ362314, OQ362316, OQ362317, OQ362315, OQ362301, OQ362345, OQ362318, OQ362341, OQ362313, OQ362308, OQ362352, OQ362346, OQ362324, OQ362325, OQ362322, OQ362321, OQ362299, OQ362329, OQ362333, OQ362335, OQ362334, OQ362298, OQ362311, OQ362323, OQ362297, OQ362305, OQ362307, OQ362296, OQ362330, OQ362300, OQ362343, OQ362331, OQ362328, OQ362303, OQ362294, OQ362347, OQ362326, OQ362306, OQ362332, OQ362309, OQ362340.
