# Supplementary material for: Genetic diversity and antagonistic properties of Trichoderma strains from the crop rhizospheres in southern Rajasthan, India
Source: Sci Rep. 2024 Apr 14;14:8610. doi: 10.1038/s41598-024-58302-5 (PMC11016547; doi:10.1038/s41598-024-58302-5)
Supplement: Supplementary file 1 — Supplementary Tables. [file 41598_2024_58302_MOESM1_ESM.docx]

**Supplementary table 1:** The isolates used in this study and their *tef-1α* GenBank Accession Numbers

| **Trichoderma species** | **Code** | **Place** | **Crop** | **Location** | **NCBI Accession no.** |
| --- | --- | --- | --- | --- | --- |
| *Trichoderma afroharzianum* | BThr1 | Chhinch, BSW | Cucumber | N23°28’17.5” E74°19’43.3” | OQ362340 |
| *Trichoderma brevicompactum* | BTbr2 | Kushalgarh, BSW | Tomato | N23°16’15.5” E74°26’31.8” | OQ362310 |
| *Trichoderma atroviride* | BTat3 | Garhi, BSW | Clusterbean | N 23⁰ 29’ 20.4”  E 74⁰3’ 12.5” | OQ362336 |
| *Trichoderma afroharzianum* | BThr4 | Dungarpur | Soybean | N23⁰46,40.1”  E 73⁰ 39’38.1” | OQ594958 |
| *Trichoderma afroharzianum* | BThr5 | ChotiSadri | Soybean | N24°26’17.1”  E74°43’8.1” | OQ362342 |
| *Trichoderma brevicompactum* | BTbr6 | Bagidora | Mungbean | N23°24’57.1” E74°16’56.6” | OQ362304 |
| *Trichoderma afroharzianum* | BThr7 | Vallabhnagar, UDR | Maize | N24⁰40’55.0”  E74⁰0’4.17” | OQ362337 |
| *Trichoderma ghanense* | BTgh8 | Pipalkhunt | Forest | N23°49’9.9”  E74°35’21.8” | OQ362349 |
| *Trichoderma brevicompactum* | BTbr10 | Kushalgarh | Sugarcane | N23°17’10.3” E74°20’35.4” | OQ362302 |
| *Trichoderma asperellum* | BTas11 | Choti Sadri, P’garh | Groundnut | N24°26’17.7”  E74°43’7.1” | OQ362312 |
| *Trichoderma* *camerunense* | BThr12 | Choti SadriP’garh | Groundnut | N24°26’17.7”  E74°43’7.1” | OQ362351 |
| *Trichoderma afroharzianum* | BThr13 | Kushalgarh, BSW | Maize | N23°12’54.8” E74°15’21.7” | OQ362339 |
| *Trichoderma afroharzianum* | BThr14 | Bagidora, BSW | Banana | N23°24’36.1” E74°16’16.1” | OQ362344 |
| *Trichoderma longibrachiatum* | BTlg15 | Ghatol, BSW | Cucumber | N23⁰40’13.5” E74⁰25’51.3” | OQ362348 |
| *Trichoderma afroharzianum* | BThr16 | Nimbahera, P’garh | Pomegranate | N24°29’55.5” E74°42’28.5” | OQ362338 |
| *Trichoderma asperellum* | BTas18 | Kushalgarh | Sugarcane | N23°17’10.3” E74°20’35.4” | OQ362320 |
| *Trichoderma inhamatum* | BThr19 | Chittorgarh | Soybean | N24°45’23.1” E74°21’17.5” | OQ362350 |
| *Trichoderma brevicompactum* | BTbr20a | Kushalgarh | Brinjal | N23°11’32.3”  E74°27’9.0” | OQ362295 |
| *Trichoderma asperellum* | BTas21 | Banswara | Tomato | N23°33’54.2” E74°45’15.2” | OQ362319 |
| *Trichoderma asperellum* | BTas23 | Choti Sadri, P’garh | Sesame | N24°23’28.5” E74°42’42.1” | OQ362314 |
| *Trichoderma asperellum* | BTas24 | Banswara | Brinjal | N23°30’53.2” E74°25’11.2” | OQ362316 |
| *Trichoderma asperellum* | BTas25 | Ghatol BSW | Cucumber | N23⁰40’13.5” E74⁰25’51.3” | OQ362317 |
| *Trichoderma asperellum* | BTas26 | Kushalgarh | Tomato | N23°11’32.3”  E74°27’9.0” | OQ362315 |
| *Trichoderma erinaceum* | BTer27 | Rajsamand | Cotton | N25°3’47.5”  E73°54’42.8” | OQ362301 |
| *Trichoderma brevicompactum* | BTbr28 | Chittorgarh | Brinjal | N23°56’29.9” E74°42’50.5” | OQ362345 |
| *Trichoderma afroharzianum* | BThr29 | Dungarpur | Maize | N23⁰41’2.9”  E 73⁰ 36’30.2” | OQ362318 |
| *Trichoderma asperellum* | BTas30 | Pratapgarh | okra | N23°56’33.0” E74°42’53.2” | OQ362341 |
| *Trichoderma afroharzianum* | BThr32 | Kushalgarh | Mungbean | N23°19’52.9” E74°18’43.6” | OQ362313 |
| *Trichoderma asperellum* | BTas33 | Kushalgarh | Mungbean | N23°19’52.9” E74°18’43.6” | OQ362308 |
| *Trichoderma brevicompactum* | BTbr34 | Sirohi | Tomato | N24°53’56.0  E73°0’5.0 | OQ362352 |
| *Trichoderma camerunense* | BThr35 | Choti Sadri | Citrus | N24°25’59.3” E74°40’36.1” | OQ362346 |
| *Trichoderma afroharzianum* | BThr37 | Kushalgarh | Sugar yam | N23°10’32.5” E74°21’49.3” | OQ362324 |
| *Trichoderma asperelloides* | BTas38 | Sirohi | Soybean | N24°23’ 41.5” E72°22’13.5” | OQ362325 |
| *Trichoderma erinaceum* | BTer39 | ChotiSadri | Pomegranate | N24°29’55.5” E74°42’28.5” | OQ362322 |
| *Trichoderma asperelloides* | BTas42 | ChotiSadri | Pomegranate | N24°20’4.5”  E74°35’59.6” | OQ362321 |
| *Trichoderma asperellum* | BTas43 | Chittorgarh | Brinjal | N23°56’33.0” E74°42’53.2” | OQ362299 |
| *Trichoderma brevicompactum* | BTbr44 | Sirohi | Clusterbean | N24°40’01.1” E72°26’37.8” | OQ362329 |
| *Trichoderma erinaceum* | BTer46 | Sirohi | Maize | N24°52’17.3”  E72°50’51.3” | OQ362333 |
| *Trichoderma erinaceum* | BTer47 | Udaipur | Maize | N24⁰33’25.6” E73⁰45’24.1” | OQ362335 |
| *Trichoderma erinaceum* | BTer48 | Kushalgarh | Tomato | N23°11’32.3”  E74°27’9.0” | OQ362334 |
| *Trichoderma erinaceum* | BTer49 | Pratapgarh | Soybean | N24°4’17.5”  E74°39’27.3” | OQ362298 |
| *Trichoderma brevicompactum* | BTbr51iv | Chittorgarh | Cotton | N24°24’15.5” E74°23’20.2” | OQ362311 |
| *Trichoderma brevicompactum* | BTbr52 | Dungarpur | Soybean | N23°54’3.2”  E73°46’03.7” | OQ362323 |
| *Trichoderma asperelloides* | BTas51 | Banswara | Maize | N23°29’0.2”  E74°12’05.9” | OQ362297 |
| *Trichoderma brevicompactum* | BTbr53 | Pipalkhunt | Forest | N23°49’36.9” E74°35’04.8” | OQ362305 |
| *Trichoderma brevicompactum* | BTbr54 | Pratapgarh | Guava | N24°10’18.5” E74°48’11.4” | OQ362307 |
| *Trichoderma brevicompactum* | BTbr55 | Dungarpur | Cucumber | N23°43’11.9” E73°44’34.7” | OQ362296 |
| *Trichoderma brevicompactum* | BTbr56 | Choti Sadri P’garh | Pomegranate | N24°17’11.3” E74°43’14.2” | OQ362330 |
| *Trichoderma erinaceum* | BTer57 | Chittorgarh | Groundnut | N24°32’22.6” E74°32’32.0” | OQ362300 |
| *Trichoderma brevicompactum* | BTbr59 | Kushalgarh, BSw | Maize | N 23⁰ 11’ 42.1”  E 74⁰16’ 3.5” | OQ362343 |
| *Trichoderma afroharzianum* | BThr60 | Rajsamand | Okra | N24⁰58’47.5” E73⁰47’11.8” | OQ362331 |
| *Trichoderma erinaceum* | BTer61 | Pratapgarh | Guava | N24°10’12.5” E74°45’16.5” | OQ362328 |
| *Trichoderma erinaceum* | Bter62 | Vallabhnagar, Udr | Soybean | N24°21’47.3” E74°18’36.6” | OQ362303 |
| *Trichoderma brevicompactum* | BTbr64a | Dungarpur | Soybean | N23°52’23.8” E73°41’16.9” | OQ362294 |
| *Trichoderma brevicompactum* | BTbr66 | Banswara | Soybean | N23°16’54.2” E74°22’15.8” | OQ362347 |
| *Trichoderma lentiforme* | BThr67 | Banswara | Paddy | N23°20’41.8” E74°30’50.1” | OQ362326 |
| *Trichoderma erinaceum* | BTer68 | Choti Sadri, P’garh | Citrus | N24°25’59.3” E74°40’36.1” | OQ362306 |
| *Trichoderma brevicompactum* | BTbr71 | Chittorgarh | Groundnut | N24°37’40.5” E74°37’12.5” | OQ362332 |
| *Trichoderma erinaceum* | BTer73 | Rajsamand | Soybean | N25°45’27.3”  E73°45’28.1” | OQ362309 |
| *Trichoderma brevicompactum* | BTbr74 | Rajsamand | Groundnut | N25°52’16.3”  E73°20’12.1” | OQ362340 |

**Supplementary table 2:**

| **Trichoderma species** | **2 DAI** | | | **4 DAI** | | | **6 DAI** | | |
| --- | --- | --- | --- | --- | --- | --- | --- | --- | --- |
|  | **PDA** | **CMA** | **TJA** | **PDA** | **CMA** | **TJA** | **PDA** | **CMA** | **TJA** |
| *T atroviride* | 1.27^de*^ | 1.4^b^ | 0.3^d^ | 3.53^e^ | 3.47^c^ | 0.63^e^ | 5.0^b^ | 4.5^b^ | 1.53^c^ |
| *T brevicompactum* | 1.0^e^ | 1.0^b^ | 0.5^cd^ | 3.93^d^ | 2.80^d^ | 1.23^d^ | 4.23^b^ | 4.23^b^ | 2.07^c^ |
| *T asperellum* | 2.23^ab^ | 2.1^a^ | 1.33^ab^ | 5.67^a^ | 4.03^bc^ | 3.23^bc^ | 7.0^a^ | 7^a^ | 5.53^ab^ |
| *T. erinaceum* | 2.63^a^ | 2.0^a^ | 1.23^ab^ | 5.33^b^ | 5.23^a^ | 3.63^ab^ | 7.0^a^ | 6.07^a^ | 5.53^ab^ |
| *T harzianum* | 2.0b^c^ | 2.0^a^ | 1.53^a^ | 5.83^a^ | 5.63^a^ | 4.0^a^ | 7.0^a^ | 7^a^ | 6.13^a^ |
| *T. ghanense* | 1.27^de^ | 1.03^b^ | 0.73^c^ | 4.13^d^ | 4.50^b^ | 2.93^c^ | 6.20^a^ | 5.97^a^ | 5.10^b^ |
| *T. hamatum* | 1.53^cd^ | 1.27^b^ | 1.13^b^ | 4.63^c^ | 4.40^b^ | 3.20^bc^ | 6.87^a^ | 6.13^a^ | 4.93^b^ |
| CV | 17.16 | 18.29 | 22.48 | 3.78 | 7.43 | 11.44 | 9.38 | 12.74 | 9.26 |
| CD (0.05) | 0.52 | 0.50 | 0.39 | 0.32 | 0.57 | 0.55 | 1.03 | 1.32 | 0.73 |

DAI= Days after incubation; PDA=Potato Dextrose Agar, CMA=Corn Meal Agar, TJA=Tomato Juice Agar; *Values are mean of the 4 replications; Means with same letter within same column are not significantly different at 5% probability level

**Supplementary table 3:** Antagonistic potential of *Trichoderma* isolates against *Sclerotium rolfsii* based on various biocontrol parameters

| Isolates | *Sclerotium rolfsii* | | | | | | | |
| --- | --- | --- | --- | --- | --- | --- | --- | --- |
|  | C | Z | M | P | Z/M | PBCI (cm^-1^) | % Inhibition |  |
| BThr1 | 2.0 | 7.3 | 10.3 | 2.5 | 0.71±0.09 | 0.56±0.01 | 63.8±3.60 |  |
| BTbr2 | 2.3 | 8.7 | 11.3 | 2.8 | 0.77±0.05 | 0.47±0.05 | 60.0±2.86 |  |
| BTat3 | 2.3 | 11.0 | 13.3 | 2.6 | 0.82±0.05 | 0.48±0.05 | 63.3±2.97 |  |
| BThr4 | 2.0 | 6.7 | 9.0 | 2.3 | 0.74±0.04 | 0.60±0.08 | 67.6±3.60 |  |
| BThr5 | 2.0 | 8.3 | 11.3 | 2.0 | 0.73±0.01 | 0.68±0.01 | 71.4±0.00 |  |
| BTbr6 | 3.0 | 10.0 | 12.7 | 2.5 | 0.79±0.05 | 0.52±0.04 | 64.8±0.82 |  |
| BThr7 | 2.0 | 8.3 | 10.7 | 2.4 | 0.78±0.05 | 0.54±0.06 | 65.7±1.43 |  |
| BTgh8 | 2.3 | 11.0 | 14.7 | 3.3 | 0.75±0.05 | 0.40±0.04 | 52.4±5.95 |  |
| BTbr10 | 2.3 | 10.7 | 12.7 | 2.5 | 0.84±0.07 | 0.49±0.12 | 64.8±5.02 |  |
| BTas11 | 2.0 | 7.7 | 10.0 | 2.2 | 0.77±0.04 | 0.60±0.11 | 68.6±4.95 |  |
| BThr12 | 2.0 | 8.7 | 13.0 | 2.4 | 0.67±0.02 | 0.64±0.05 | 66.2±2.18 |  |
| BThr13 | 2.0 | 7.3 | 10.0 | 2.9 | 0.73±0.06 | 0.48±0.03 | 59.0±1.65 |  |
| BThr14 | 2.3 | 7.3 | 9.3 | 2.3 | 0.79±0.09 | 0.59±0.19 | 67.6±7.87 |  |
| BTlg15 | 1.7 | 10.0 | 13.3 | 2.8 | 0.75±0.02 | 0.48±0.04 | 60.5±3.60 |  |
| BThr16 | 2.0 | 7.3 | 9.3 | 2.0 | 0.79±0.01 | 0.63±0.08 | 71.0±3.60 |  |
| BTas18 | 2.3 | 10.3 | 13.0 | 3.0 | 0.79±0.04 | 0.42±0.08 | 56.7±5.95 |  |
| BThr19 | 2.0 | 7.3 | 9.3 | 2.9 | 0.79±0.01 | 0.45±0.03 | 59.0±2.18 |  |
| BTbr20 | 3.0 | 8.0 | 11.0 | 3.1 | 0.73±0.04 | 0.44±0.05 | 55.7±5.15 |  |
| BTas21 | 2.3 | 7.0 | 9.3 | 1.9 | 0.75±0.05 | 0.70±0.07 | 72.4±4.59 |  |
| BTas23 | 2.3 | 6.3 | 8.7 | 2.7 | 0.73±0.06 | 0.51±0.07 | 61.0±3.60 |  |
| BTas24 | 2.3 | 8.0 | 10.7 | 2.2 | 0.75±0.06 | 0.61±0.11 | 68.1±3.60 |  |
| BTas25 | 2.3 | 7.7 | 10.0 | 2.0 | 0.77±0.04 | 0.65±0.03 | 71.4±0.00 |  |
| BTas26 | 2.3 | 7.3 | 9.7 | 2.5 | 0.76±0.05 | 0.52v0.06 | 63.8±3.60 |  |
| BTer27 | 2.3 | 8.3 | 10.7 | 2.1 | 0.78±0.05 | 0.61±0.10 | 69.5±4.59 |  |
| BTbr28 | 2.3 | 9.3 | 13.3 | 2.8 | 0.70±0.13 | 0.52±0.11 | 60.0±5.15 |  |
| BThr29 | 2.0 | 5.7 | 7.7 | 1.8 | 0.74±0.02 | 0.77±0.02 | 74.8±0.82 |  |
| BTas30 | 2.0 | 8.0 | 10.3 | 2.5 | 0.78±0.03 | 0.51±0.03 | 63.8±3.60 |  |
| BThr32 | 2.0 | 8.3 | 10.0 | 2.4 | 0.84±0.05 | 0.51±0.02 | 66.2±1.65 |  |
| BTas33 | 2.3 | 11.0 | 14.7 | 2.4 | 0.75±0.04 | 0.55±0.01 | 65.2±1.65 |  |
| BTbr34 | 2.3 | 8.7 | 12.0 | 2.9 | 0.72±0.10 | 0.51±0.16 | 58.6±10.30 |  |
| BThr35 | 2.3 | 7.3 | 10.0 | 2.6 | 0.74±0.04 | 0.52±0.07 | 62.4±3.30 |  |
| BThr37 | 2.0 | 7.0 | 9.3 | 2.3 | 0.75±0.07 | 0.60±0.14 | 67.1±4.29 |  |
| BTas38 | 2.0 | 7.3 | 9.7 | 2.4 | 0.76±0.08 | 0.57±0.14 | 65.2±8.61 |  |
| BTer39 | 1.7 | 15.3 | 18.0 | 3.0 | 0.85±0.02 | 0.39±0.00 | 57.1±1.43 |  |
| BTas42 | 2.0 | 7.0 | 9.0 | 2.3 | 0.78±0.03 | 0.55±0.01 | 66.7±0.82 |  |
| BTas43 | 2.0 | 7.7 | 9.7 | 2.5 | 0.79±0.02 | 0.53±0.10 | 64.8±6.60 |  |
| BTbr44 | 2.0 | 8.7 | 12.7 | 2.9 | 0.70±0.09 | 0.50±0.07 | 58.6±1.43 |  |
| BTer46 | 2.3 | 11.7 | 14.3 | 3.0 | 0.81±0.06 | 0.46±0.17 | 57.1±16.23 |  |
| BTer47 | 2.0 | 11.3 | 14.0 | 2.8 | 0.81±0.10 | 0.45±0.01 | 60.5±5.41 |  |
| BTer48 | 2.3 | 8.7 | 11.3 | 2.7 | 0.76±0.06 | 0.49±0.08 | 61.4±3.78 |  |
| BTer49 | 3.0 | 9.3 | 11.7 | 2.7 | 0.80±0.06 | 0.48±0.10 | 61.9±6.75 |  |
| BTbr51 | 2.3 | 10.7 | 13.7 | 3.1 | 0.77±0.05 | 0.42±0.07 | 55.7±5.15 |  |
| BTbr52 | 2.3 | 9.0 | 13.7 | 3.5 | 0.67±0.11 | 0.44±0.07 | 49.5±6.60 |  |
| BTas51 | 2.0 | 11.0 | 14.3 | 3.4 | 0.76±0.06 | 0.38±0.03 | 51.0±1.65 |  |
| BTbr53 | 2.0 | 11.0 | 13.7 | 3.2 | 0.81±0.04 | 0.39±0.02 | 54.3±1.43 |  |
| BTbr54 | 2.3 | 11.0 | 14.3 | 3.0 | 0.77±0.05 | 0.46±0.12 | 57.1±11.16 |  |
| BTbr55 | 2.3 | 7.7 | 10.3 | 2.7 | 0.74±0.03 | 0.50±0.05 | 61.4±3.78 |  |
| BTbr56 | 2.3 | 11.0 | 14.7 | 3.3 | 0.75±0.05 | 0.46±0.19 | 52.4±24.43 |  |
| BTer57 | 2.3 | 9.7 | 12.3 | 3.0 | 0.78±0.02 | 0.42±0.04 | 56.7±3.60 |  |
| BTbr59 | 2.3 | 11.7 | 15.3 | 3.1 | 0.76±0.08 | 0.44±0.09 | 56.2±4.36 |  |
| BThr60 | 2.3 | 7.7 | 10.3 | 3.1 | 0.74±0.05 | 0.43±0.04 | 55.2±3.30 |  |
| BTer61 | 2.3 | 10.7 | 13.0 | 2.9 | 0.82±0.06 | 0.43±0.07 | 59.0±5.77 |  |
| BTer62 | 2.3 | 10.3 | 12.7 | 2.4 | 0.81±0.06 | 0.52±0.05 | 66.2±1.65 |  |
| BTbr64 | 2.0 | 10.7 | 14.0 | 3.6 | 0.76±0.03 | 0.37±0.03 | 49.0±5.77 |  |
| BTbr66 | 2.3 | 11.0 | 14.0 | 3.5 | 0.79±0.06 | 0.36±0.05 | 49.5±3.60 |  |
| BThr67 | 3.0 | 13.7 | 16.7 | 3.9 | 0.82±0.06 | 0.32±0.05 | 44.8±5.77 |  |
| BTer68 | 1.7 | 8.7 | 11.7 | 3.2 | 0.74±0.08 | 0.44±0.11 | 54.3±7.56 |  |
| BTbr71 | 2.0 | 10.3 | 13.7 | 3.2 | 0.76±0.02 | 0.41±0.02 | 53.8±3.60 |  |
| BTer73 | 2.0 | 11.3 | 14.3 | 2.7 | 0.79±0.06 | 0.49±0.14 | 61.0±9.72 |  |
| BTbr74 | 2.3 | 9.0 | 11.3 | 3.2 | 0.79±0.06 | 0.40±0.09 | 54.3±7.56 |  |
| CV | 15.31 | 10.85 | 9.56 | 12.59 | 7.25 | 13.04 | 7.89 |  |
| CD at 1% | 0.714 | 2.089 | 2.397 | 0.723 | 0.00 | 0.138 | 10.1 |  |
| CD at 5% | 0.543 | 1.59 | 1.824 | 0.55 | 0.09 | 0.105 | 7.69 |  |

C = days required for the two antagonist colonies to come into contact; Z = days required for the BCA to fully grow over the pathogen colony; M = days required for the BCA to fully grow over the plate; P = the radial growth distance (in cm) of pathogen colony between the point of inoculation and the marginal point of contact with the BCA growth zone; R = the pathogen resistance index to the BCA was defined as a ratio of Z/M based on the periods required for the full growth of a fungal BCA in the presence (Z) and absence (M) of pathogen; PBCI = Pakdaman’s biological control index.

**Supplementary table 4:** Antagonistic potential of *Trichoderma* isolates against *Rhizoctonia solani* based on various biocontrol parameters

| **Isolates** | ***Rhizoctonia solani*** | | | | | | | |
| --- | --- | --- | --- | --- | --- | --- | --- | --- |
|  | **C** | **Z** | **M** | **P** | **Z/M** | **PBCI (cm ^-1^)** | **% Inhibition** |  |
| BThr1 | 1 | 7.3 | 8.3 | 1.9 | 0.88±0.01 | 0.68±0.10 | 75.7±3.78 |  |
| BTbr2 | 2 | 6.3 | 8.0 | 2.5 | 0.79±0.07 | 0.50±0.03 | 63.8±3.60 |  |
| BTat3 | 2 | 5.7 | 7.3 | 1.7 | 0.77±0.07 | 0.76±0.05 | 75.7±1.43 |  |
| BThr4 | 2 | 5.3 | 6.7 | 1.6 | 0.80±0.08 | 0.81±0.20 | 77.1±5.15 |  |
| BThr5 | 2 | 5.0 | 6.3 | 1.7 | 0.79±0.07 | 0.75±0.07 | 75.7±2.47 |  |
| BTbr6 | 2 | 6.3 | 8.0 | 1.9 | 0.79±0.06 | 0.65±0.01 | 72.4±1.65 |  |
| BThr7 | 2 | 6.0 | 7.3 | 1.9 | 0.82±0.06 | 0.68±0.19 | 73.3±5.77 |  |
| BTgh8 | 3 | 7.0 | 9.3 | 2.3 | 0.76±0.12 | 0.57±0.06 | 66.7±4.12 |  |
| BTbr10 | 2 | 5.7 | 7.3 | 1.9 | 0.77±0.07 | 0.69±0.08 | 72.9±1.43 |  |
| BTas11 | 2 | 5.0 | 6.3 | 1.9 | 0.79±0.07 | 0.67±0.09 | 72.9±1.43 |  |
| BThr12 | 2 | 5.0 | 6.3 | 2.4 | 0.79±0.07 | 0.52±0.04 | 65.2±1.65 |  |
| BThr13 | 2 | 3.7 | 5.7 | 2.1 | 0.64±0.04 | 0.73±0.04 | 69.5±1.65 |  |
| BThr14 | 2 | 5.7 | 7.0 | 2.3 | 0.81±0.06 | 0.54±0.03 | 67.1±2.86 |  |
| BTlg15 | 2 | 7.7 | 10.0 | 2.3 | 0.77±0.06 | 0.58±0.07 | 67.6±3.60 |  |
| BThr16 | 2 | 5.0 | 6.3 | 1.9 | 0.79±0.07 | 0.68±0.09 | 73.3±1.65 |  |
| BTas18 | 2 | 6.3 | 7.7 | 2.4 | 0.82±0.09 | 0.52±0.13 | 65.7±3.78 |  |
| BThr19 | 2 | 6.0 | 8.0 | 2.1 | 0.77±0.15 | 0.63±0.11 | 70.0±1.43 |  |
| BTbr20 | 2 | 6.7 | 8.3 | 2.4 | 0.80±0.07 | 0.52±0.06 | 65.2±1.65 |  |
| BTas21 | 2 | 6.0 | 7.7 | 2.1 | 0.79±0.06 | 0.62±0.11 | 70.0±5.15 |  |
| BTas23 | 2 | 6.0 | 7.7 | 2.4 | 0.79±0.06 | 0.54±0.11 | 65.2±5.77 |  |
| BTas24 | 2 | 6.3 | 7.3 | 2.2 | 0.87±0.13 | 0.54±0.09 | 69.0±2.18 |  |
| BTas25 | 2 | 6.3 | 7.7 | 1.2 | 0.83±0.07 | 1.06±0.24 | 83.3±2.18 |  |
| BTas26 | 2 | 5.3 | 7.3 | 1.9 | 0.74±0.10 | 0.74±0.07 | 73.3±1.65 |  |
| BTer27 | 2 | 5.3 | 8.0 | 2.1 | 0.67±0.07 | 0.73±0.20 | 69.5±5.77 |  |
| BTbr28 | 2 | 6.0 | 7.7 | 2.4 | 0.78±0.08 | 0.55±0.04 | 66.2±1.65 |  |
| BThr29 | 2 | 5.7 | 6.7 | 1.1 | 0.85±0.01 | 1.11±0.12 | 84.8±1.65 |  |
| BTas30 | 2 | 5.7 | 7.7 | 2.2 | 0.77±0.07 | 0.60±0.10 | 69.0±2.18 |  |
| BThr32 | 2 | 6.3 | 7.7 | 1.6 | 0.83±0.07 | 0.78±0.04 | 77.6±2.97 |  |
| BTas33 | 1 | 5.3 | 6.3 | 2.0 | 0.84±0.01 | 0.59±0.07 | 71.0±3.60 |  |
| BTbr34 | 2 | 6.7 | 8.7 | 2.1 | 0.77±0.03 | 0.62±0.01 | 70.0±1.43 |  |
| BThr35 | 2 | 8.0 | 11.0 | 2.0 | 0.73±0.00 | 0.69±0.12 | 71.0±5.77 |  |
| BThr37 | 2 | 7.3 | 9.3 | 2.2 | 0.79±0.09 | 0.59±0.06 | 69.0±2.18 |  |
| BTas38 | 2 | 5.0 | 6.7 | 1.8 | 0.75±0.07 | 0.76±0.05 | 74.8±3.60 |  |
| BTer39 | 1 | 6.7 | 8.0 | 2.1 | 0.83±0.07 | 0.61±0.15 | 70.5±7.33 |  |
| BTas42 | 2 | 5.3 | 7.0 | 1.9 | 0.76±0.08 | 0.71±0.07 | 73.3±1.65 |  |
| BTas43 | 1 | 7.0 | 8.3 | 1.3 | 0.84±0.06 | 0.92±0.13 | 81.4±1.43 |  |
| BTbr44 | 2 | 7.0 | 9.0 | 1.8 | 0.78±0.09 | 0.72±0.15 | 74.3±2.86 |  |
| BTer46 | 2 | 5.0 | 7.0 | 1.9 | 0.72±0.10 | 0.78±0.25 | 73.3±4.59 |  |
| BTer47 | 2 | 6.7 | 9.0 | 1.6 | 0.74±0.04 | 0.87±0.20 | 77.1±3.78 |  |
| BTer48 | 2 | 8.0 | 10.3 | 1.5 | 0.77±0.05 | 0.91±0.30 | 78.6±5.15 |  |
| BTer49 | 2 | 7.3 | 9.7 | 2.1 | 0.76±0.05 | 0.63±0.05 | 70.0±1.43 |  |
| BTbr51 | 2 | 9.7 | 12.0 | 2.3 | 0.81±0.05 | 0.55±0.07 | 67.6±2.97 |  |
| BTbr52 | 2 | 7.0 | 8.7 | 2.3 | 0.81±0.04 | 0.55±0.03 | 67.6±3.60 |  |
| BTas51 | 2 | 7.0 | 9.0 | 2.1 | 0.78±0.03 | 0.62±0.02 | 70.5±1.64 |  |
| BTbr53 | 2 | 7.0 | 9.3 | 2.0 | 0.75±0.07 | 0.66±0.08 | 71.0±0.82 |  |
| BTbr54 | 2 | 7.0 | 9.0 | 2.4 | 0.78±0.11 | 0.57±0.17 | 66.2±5.77 |  |
| BTbr55 | 2 | 7.0 | 9.0 | 2.0 | 0.77±0.05 | 0.64±0.05 | 71.0±0.82 |  |
| BTbr56 | 2 | 7.3 | 10.0 | 2.5 | 0.73±0.06 | 0.56±0.07 | 64.8±2.18 |  |
| BTer57 | 2 | 7.0 | 9.7 | 2.0 | 0.73±0.04 | 0.68±0.03 | 71.0±2.18 |  |
| BTbr59 | 2 | 6.3 | 8.3 | 2.3 | 0.76±0.02 | 0.57±0.04 | 66.7±2.97 |  |
| BThr60 | 2 | 5.3 | 7.7 | 2.1 | 0.70±0.06 | 0.69±0.08 | 69.5±5.95 |  |
| BTer61 | 2 | 8.3 | 10.7 | 2.2 | 0.78±0.05 | 0.58±0.10 | 68.1±3.60 |  |
| BTer62 | 2 | 9.7 | 13.0 | 1.9 | 0.75±0.07 | 0.71±0.08 | 72.9±1.43 |  |
| BTbr64 | 2 | 8.0 | 9.7 | 2.2 | 0.83±0.07 | 0.56±0.08 | 69.0±2.18 |  |
| BTbr66 | 2 | 9.7 | 12.3 | 2.4 | 0.78±0.00 | 0.54±0.03 | 66.2±1.65 |  |
| BThr67 | 3 | 8.3 | 10.7 | 2.2 | 0.78±0.05 | 0.58±0.10 | 68.1±3.60 |  |
| BTer68 | 2 | 10.7 | 13.0 | 2.2 | 0.82±0.02 | 0.57±0.12 | 68.6±7.56 |  |
| BTbr71 | 2 | 8.3 | 11.0 | 1.9 | 0.76±0.05 | 0.68±0.00 | 72.4±1.65 |  |
| BTer73 | 2 | 8.7 | 11.0 | 2.7 | 0.79±0.05 | 0.48±0.06 | 61.9±5.95 |  |
| BTbr74 | 2 | 6.7 | 8.3 | 2.3 | 0.80±0.07 | 0.55±0.11 | 66.7±5.95 |  |
| CV | 5.27 | 11.92 | 11.473 | 11.09 | 7.84 | 13.65 | 6.51 |  |
| CD at 1% | 0.224 | 1.668 | 2.061 | 0.477 |  | 0.189 | 4.276 |  |
| CD at 5% | 0.17 | 1.269 | 1.568 | 0.363 | 0.098 | 0.144 | 3.253 |  |

C = days required for the two antagonist colonies to come into contact; Z = days required for the BCA to fully grow over the pathogen colony; M = days required for the BCA to fully grow over the plate; P = the radial growth distance (in cm) of pathogen colony between the point of inoculation and the marginal point of contact with the BCA growth zone; R = the pathogen resistance index to the BCA was defined as a ratio of Z/M based on the periods required for the full growth of a fungal BCA in the presence (Z) and absence (M) of pathogen; PBCI = Pakdaman’s biological control index.

**Supplementary table 5:** Antagonistic potential of *Trichoderma* isolates against *Fusarium verticillioides* based on various biocontrol parameters

| **Isolates** | ***Fusarium verticiliioides*** | | | | | | | |
| --- | --- | --- | --- | --- | --- | --- | --- | --- |
|  | **C** | **Z** | **M** | **P** | **Z/M** | **PBCI cm ^-1^** | **%**  **Inhibition** |  |
| BThr1 | 2.0 | 6.0 | 7.3 | 1.3 | 0.82±0.06 | 1.07±0.41 | 80.5±9.51 |  |
| BTbr2 | 2.0 | 5.3 | 6.3 | 1.3 | 0.84±0.04 | 0.99±0.27 | 80.6±6.48 |  |
| BTat3 | 2.0 | 6.0 | 8.0 | 1.2 | 0.75±0.13 | 1.14±0.35 | 82.4±3.30 |  |
| BThr4 | 2.0 | 5.7 | 7.0 | 1.2 | 0.81±0.08 | 1.08±0.16 | 83.3±2.18 |  |
| BThr5 | 1.7 | 5.7 | 7.3 | 1.3 | 0.77±0.07 | 1.06±0.37 | 81.4±4.95 |  |
| BTbr6 | 2.0 | 5.0 | 6.7 | 0.8 | 0.75±0.07 | 1.76±0.57 | 88.6±2.86 |  |
| BThr7 | 1.7 | 6.0 | 7.3 | 0.8 | 0.82±0.09 | 1.77±0.65 | 89.0±4.12 |  |
| BTgh8 | 2.0 | 7.3 | 8.3 | 1.2 | 0.88±0.01 | 1.01±0.33 | 82.4±7.05 |  |
| BTbr10 | 1.7 | 5.3 | 7.3 | 1.0 | 0.73±0.12 | 1.39±0.22 | 85.7±0.00 |  |
| BTas11 | 2.7 | 5.7 | 7.0 | 1.1 | 0.81±0.06 | 1.19±0.46 | 83.8±5.95 |  |
| BThr12 | 1.7 | 5.0 | 6.3 | 0.9 | 0.79±0.07 | 1.38±0.32 | 86.7±1.65 |  |
| BThr13 | 2.0 | 5.7 | 7.0 | 0.9 | 0.81±0.08 | 1.39±0.39 | 86.7±3.30 |  |
| BThr14 | 2.3 | 10.3 | 11.3 | 1.2 | 0.90±0.03 | 1.00±0.25 | 83.3±5.41 |  |
| BTlg15 | 1.7 | 8.0 | 10.0 | 1.5 | 0.81±0.07 | 0.83±0.17 | 78.1±3.60 |  |
| BThr16 | 1.7 | 5.3 | 6.7 | 1.1 | 0.80±0.08 | 1.14±0.07 | 84.3±1.43 |  |
| BTas18 | 2.3 | 10.0 | 11.3 | 1.0 | 0.88±0.06 | 1.12±0.15 | 85.2±2.18 |  |
| BThr19 | 2.7 | 8.3 | 9.3 | 1.1 | 0.89±0.03 | 1.06±0.12 | 84.8±1.65 |  |
| BTbr20 | 2.0 | 6.7 | 8.3 | 1.4 | 0.80±0.07 | 0.88±0.11 | 79.5±1.65 |  |
| BTas21 | 2.0 | 6.0 | 7.7 | 1.0 | 0.79±0.04 | 1.34±0.36 | 85.7±3.78 |  |
| BTas23 | 1.7 | 5.3 | 7.0 | 1.1 | 0.77±0.06 | 1.17±0.17 | 83.8±1.65 |  |
| BTas24 | 1.7 | 6.0 | 7.3 | 1.1 | 0.82±0.06 | 1.14±0.35 | 83.8±4.59 |  |
| BTas25 | 1.7 | 5.3 | 6.7 | 1.0 | 0.81±0.05 | 1.30±0.17 | 86.2±2.18 |  |
| BTas26 | 1.7 | 5.7 | 7.0 | 1.0 | 0.81±0.06 | 1.31±0.27 | 86.2±2.97 |  |
| BTer27 | 2.3 | 9.3 | 10.7 | 1.2 | 0.88±0.02 | 1.01±0.22 | 83.3±4.12 |  |
| BTbr28 | 2.3 | 10.3 | 12.0 | 1.1 | 0.87±0.04 | 1.07±0.29 | 83.8±4.59 |  |
| BThr29 | 1.7 | 4.7 | 6.0 | 0.7 | 0.78±0.10 | 1.83±0.58 | 89.5±1.65 |  |
| BTas30 | 1.7 | 5.7 | 7.0 | 0.8 | 0.77±0.03 | 1.68±0.54 | 88.1±3.60 |  |
| BThr32 | 2.0 | 7.0 | 8.3 | 1.1 | 0.84±0.04 | 1.05±0.11 | 83.8±1.65 |  |
| BTas33 | 2.7 | 6.0 | 7.7 | 1.1 | 0.79±0.14 | 1.27±0.45 | 84.8±2.97 |  |
| BTbr34 | 2.0 | 10.7 | 12.3 | 1.2 | 0.86±0.09 | 0.97±0.18 | 82.4±3.60 |  |
| BThr35 | 3.0 | 5.3 | 6.3 | 1.1 | 0.84±0.01 | 1.12±0.11 | 84.8±1.65 |  |
| BThr37 | 1.7 | 5.7 | 7.0 | 1.3 | 0.81±0.06 | 0.99±0.19 | 81.9±2.97 |  |
| BTas38 | 2.0 | 6.3 | 7.3 | 1.0 | 0.86±0.01 | 1.16±0.01 | 85.7±0.00 |  |
| BTer39 | 1.7 | 6.0 | 8.0 | 1.1 | 0.75±0.13 | 1.37±0.62 | 84.3±5.15 |  |
| BTas42 | 2.0 | 6.0 | 7.3 | 0.9 | 0.82±0.06 | 1.55±0.62 | 87.6±4.36 |  |
| BTas43 | 2.3 | 6.3 | 7.3 | 1.1 | 0.86±0.01 | 1.14±0.42 | 83.8±7.05 |  |
| BTbr44 | 1.7 | 6.7 | 8.3 | 1.0 | 0.80±0.03 | 1.22±0.20 | 85.2±2.18 |  |
| BTer46 | 2.0 | 5.3 | 6.7 | 1.3 | 0.79±0.11 | 1.06±0.42 | 81.4±5.15 |  |
| BTer47 | 2.0 | 5.7 | 7.0 | 1.4 | 0.81±0.08 | 0.97±0.35 | 80.0±6.55 |  |
| BTer48 | 1.7 | 6.0 | 8.0 | 1.4 | 0.75±0.13 | 1.07±0.47 | 80.5±4.59 |  |
| BTer49 | 1.7 | 6.0 | 7.7 | 1.2 | 0.78±0.08 | 1.25±0.69 | 82.4±7.87 |  |
| BTbr51 | 1.7 | 6.0 | 7.3 | 1.3 | 0.82±0.09 | 0.99±0.37 | 81.0±5.02 |  |
| BTbr52 | 2.0 | 6.7 | 7.7 | 1.1 | 0.87±0.01 | 1.07±0.27 | 84.0±3.38 |  |
| BTas51 | 2.3 | 6.0 | 7.0 | 1.1 | 0.86±0.00 | 1.26±0.48 | 85.0±6.89 |  |
| BTbr53 | 2.7 | 8.0 | 9.3 | 1.2 | 0.86±0.04 | 1.13±0.54 | 83.3±6.44 |  |
| BTbr54 | 2.0 | 7.0 | 9.0 | 0.8 | 0.79±0.07 | 1.57±0.35 | 88.1±2.18 |  |
| BTbr55 | 2.0 | 5.3 | 6.7 | 1.2 | 0.80±0.05 | 1.06±0.24 | 82.9±2.47 |  |
| BTbr56 | 2.7 | 7.7 | 9.0 | 1.4 | 0.85±0.06 | 0.83±0.10 | 79.5±2.97 |  |
| BTer57 | 2.0 | 7.0 | 8.3 | 1.5 | 0.84±0.06 | 0.78±0.07 | 78.1±0.82 |  |
| BTbr59 | 2.3 | 7.0 | 8.3 | 1.5 | 0.84±0.06 | 0.81±0.09 | 79.0±0.82 |  |
| BThr60 | 1.7 | 4.3 | 5.3 | 1.3 | 0.81±0.02 | 0.96±0.14 | 81.4±2.47 |  |
| BTer61 | 1.7 | 5.7 | 7.0 | 1.2 | 0.81±0.06 | 1.04±0.25 | 82.4±3.60 |  |
| BTer62 | 1.7 | 6.3 | 7.7 | 1.1 | 0.83±0.05 | 1.33±0.74 | 84.3±7.95 |  |
| BTbr64 | 2.3 | 6.3 | 8.0 | 1.2 | 0.80±0.12 | 1.04±0.11 | 82.4±3.60 |  |
| BTbr66 | 2.3 | 8.0 | 10.0 | 1.3 | 0.82±0.11 | 1.11±0.61 | 81.9±6.44 |  |
| BThr67 | 2.3 | 6.7 | 8.3 | 1.5 | 0.81±0.12 | 0.86±0.26 | 78.6±2.86 |  |
| BTer68 | 2.0 | 6.0 | 8.0 | 1.2 | 0.75±0.13 | 1.22±0.52 | 82.9±4.29 |  |
| BTbr71 | 2.7 | 7.3 | 9.3 | 1.4 | 0.79±0.07 | 0.92±0.21 | 80.0±2.86 |  |
| BTer73 | 5.3 | 7.0 | 8.3 | 1.5 | 0.84±0.06 | 0.84±0.21 | 79.0±3.60 |  |
| BTbr74 | 2.7 | 6.7 | 8.7 | 1.7 | 0.78±0.08 | 0.79±0.15 | 76.2±2.18 |  |
| CV | 25.36 | 19.98 | 16.61 | 17.52 | 7.166 | 21 | 3.64 |  |
| CD 1% | 1.114 | 2.729 | 2.778 | 0.431 | NS | 0.508 | 6.37 |  |
| CD 5% | 0.848 | 2.076 | 2.114 | 0.328 | NS | 0.386 | 4.85 |  |

C = days required for the two antagonist colonies to come into contact; Z = days required for the BCA to fully grow over the pathogen colony; M = days required for the BCA to fully grow over the plate; P = the radial growth distance (in cm) of pathogen colony between the point of inoculation and the marginal point of contact with the BCA growth zone; R = the pathogen resistance index to the BCA was defined as a ratio of Z/M based on the periods required for the full growth of a fungal BCA in the presence (Z) and absence (M) of pathogen; PBCI = Pakdaman’s biological control index.
